# Candidate Effectors From *Uromyces appendiculatus*, the Causal Agent of Rust on Common Bean, Can Be Discriminated Based on Suppression of Immune Responses

**DOI:** 10.3389/fpls.2019.01182

**Published:** 2019-10-04

**Authors:** Mingsheng Qi, Yu Mei, James P. Grayczyk, Luana M. Darben, Martin E. G. Rieker, Janina M. Seitz, Ralf T. Voegele, Steven A. Whitham, Tobias I. Link

**Affiliations:** ^1^Department of Plant Pathology and Microbiology, Iowa State University, Ames, IA, United States; ^2^Plant Biotechnology, Embrapa Soja, Londrina, Brazil; ^3^Institut für Phytomedizin, Universität Hohenheim, Stuttgart, Germany

**Keywords:** *Uromyces appendiculatus*, effectors, PTI suppression, ETI suppression, bax cell death suppression, localization, expression patterns

## Abstract

Rust fungi are devastating pathogens for several important crop plants. The biotrophic lifestyle of rust fungi requires that they influence their host plants to create a favorable environment for growth and reproduction. Rust fungi secrete a variety of effector proteins that manipulate host target proteins to alter plant metabolism and suppress defense responses. Because of the obligate biotrophic lifestyle of rust fungi, direct evidence for effector function is difficult to obtain, and so suites of experiments utilizing expression in heterologous systems are necessary. Here, we present results from a yeast cell death suppression assay and assays for suppression of PAMP-triggered immunity (PTI) and effector triggered immunity (ETI) based on delivery of effectors through the bacterial type III secretion system. In addition, subcellular localization was tested using transient expression of GFP fusion proteins in *Nicotiana benthamiana* through *Agrobacterium* infiltration. We tested 31 representative effector candidates from the devastating common bean rust pathogen *Uromyces appendiculatus*. These effector candidates were selected based on features of their gene families, most important lineage specificity. We show that several of our effector candidates suppress plant defense. Some of them also belong to families of effector candidates that are present in multiple rust species where their homologs probably also have effector functions. In our analysis of candidate effector mRNA expression, some of those effector candidates that gave positive results in the other assays were not up-regulated during plant infection, indicating that either these proteins have functions at multiple life stages or that strong up-regulation of RNA level *in planta* may not be as important a criterion for identifying effectors as previously thought. Overall, our pipeline for selecting effector candidates based on sequence features followed by screening assays using heterologous expression systems was successful in discriminating effector candidates. This work lays the foundation for functional characterization of *U. appendiculatus* effectors, the identification of effector targets, and identification of novel sources for resistance in common bean.

## Introduction

Fungal pathogens use effectors to influence their host plants. These effectors may function in weakening or killing the infected tissue as is the case for necrotrophic pathogens. Alternatively, effectors produced by biotrophic pathogens may influence the host into transferring nutrients or forming structures to accommodate the pathogen. Perhaps, the most important among the effectors are those that suppress plant defense responses. Suppression of plant defense is especially important for biotrophic pathogens since they can be effectively killed by hypersensitive plant cell death responses.

Recently, we described a comprehensive pipeline of assays in heterologous systems that aimed to identify true effectors from collections of candidate effectors (Qi et al., 2018). This pipeline is centered on identifying candidate effectors that suppress plant defense responses. Due to their biotrophic lifestyle, it is still impossible to genetically transform rust fungi with a single exception that is based on a cloned avirulence gene (Lawrence et al., 2010), which makes it necessary to utilize heterologous organisms to test for defense suppression. The bacterial type III secretion system (T3SS), which has the natural purpose of transferring effector proteins into host cells, is a well-established approach for testing the ability of fungal effectors to suppress defense responses (Sohn et al., 2007). To induce transfer of a fungal protein *via* the T3SS, a plasmid is introduced that fuses the T3SS signal peptide to the N-terminus of the fungal candidate effector replacing its own signal peptide. In our assays, we used *Pseudomonas syringae* pv. *tomato* strain DC3000 (*Pst* DC3000) or *Pseudomonas fluorescens* effector to host analysis (EtHAn) (a non-pathogen with artificially added T3SS) (Thomas et al., 2009) to deliver candidate effectors into *Nicotiana benthamiana*. *Pst* DC3000 causes HR when it is infiltrated to *N. benthamiana*—except if it carries an effector that can suppress HR. On the other hand, *P. fluorescens* triggers basal defense. Evidence for the basal defense reaction is provided by infiltration with *Pst* DC3000 several hours later; where basal defense is already established, *Pst* DC3000 cannot trigger a subsequent HR (Oh and Collmer, 2005). Therefore, if leaf tissue, which was first infiltrated with *P. fluorescens* EtHAn carrying an effector candidate and later with *Pst* DC3000, shows HR, this can be considered evidence that the effector can suppress basal defense.

Because suppression of HR can be connected to suppression of cell death, we complemented our defense suppression assays with a cell death suppression assay in yeast. For this, we used a yeast system with galactose inducible BAX-induced cell death. If yeast cells expressing a candidate effector grow well on a medium containing galactose the effector suppresses cell death in this assay. Subcellular localization can also hint at effector function (Petre et al., 2015; Petre et al., 2016). We used agroinfiltration to transiently express GFP fusion proteins of our effector candidates in *N. benthamiana*. Another indicator of effector function is expression of the effector candidate that coincides with the potential function. For those candidate effectors that yielded interesting results in the screens described above, we also elucidated the expression pattern throughout the infection process starting with the urediospore.

In previous work (Link et al., 2014; Qi et al., 2016; Qi et al., 2018), we aimed to identify effectors of *Phakopsora pachyrhizi*, which causes Asian soybean rust. Here, we used a similar pipeline to assess a collection of effector candidates from *Uromyces appendiculatus*. The effector candidates were selected from haustorial-expressed proteins that were predicted to encode a signal peptide (Link et al., 2014). *U. appendiculatus* is the causal agent of common bean rust, which is one of the most devastating diseases of common bean (*Phaseolus vulgaris*). Common bean rust leads to severe yield losses through reduced photosynthesis in the infected leaves and the carbohydrate sink formed by the fungus resulting in reduced numbers of pods, reduced pod fill, and therefore, fewer seeds (Liebenberg and Pretorius, 2010). Common bean is an important protein source for millions of people in Mesoamerica and Africa where it is cultivated in small plots for self-supply. Although resistance genes are available to be deployed against *U. appendiculatus* (Souza et al., 2008), gene-for-gene resistance has often been overcome by rust pathogens, so it cannot be considered as durable. Novel approaches to achieve resistance with improved durability may revolve around effectors. For example, the expression of effectors could be silenced using host-induced gene silencing (HIGS), effector targets in the host plant could be deleted or modified to render effectors ineffective or artificial resistance proteins interacting with the effectors could be introduced into plants. In our study on haustorial transcriptomes (Link et al., 2014), we explored *P. pachyrhizi* and *U. appendiculatus* together, expecting to find differences and commonalities in the effector repertoire. The same expectation was yet another reason for us to continue research on the *U. appendiculatus* effector candidates. In the meantime, additional information on potential *U. appendiculatus* effectors has been obtained using proteomics and HIGS approaches (Cooper et al., 2016; Cooper and Campbell, 2017). Some of these candidates were investigated by us as well, and where possible, a comparison of results is presented.

## Materials and Methods

### Bacterial Strains and Plasmids Mobilizations

Bacterial strains used in this study are listed in **Table 1**. *E. coli* and *Agrobacterium tumefaciens* were grown in Lysogeny broth (LB). *Pseudomonas* strains were grown in King’s B (KB) medium at 28°C. Standard triparental mating with *E. coli* HB101 (pRK2013) as a helper strain was used to mobilize plasmids from *E. coli* to *Pseudomonas* strains.

**Table 1:** Strains and plasmids.

Strain or plasmid	Genotype or relevant phenotype*	Source or reference
*E. coli*		
DH5α	F- *end*A1 *gln*V44 *thi*-1 *rec*A1 *rel*A1 *gyr*A96 *deo*R nupG Φ80d*lacZ*ΔM15 Δ(띘*lacZYA-argF*)U169 *hsd*R17(r_K_−m_K_+) λ^–^	Invitrogen
TOP10	F- *mcr*A Δ(띘*mrr-hsdRMS-mcrBC*) Φ80*lacZ*ΔM15 Δ*lacX74* *nup*G *rec*A1 *ara*D139 Δ(띘*ara-leu*)7697 *gal*E15 *gal*K16 *rps*L(Spr) *end*A1 λ-	Invitrogen
*P. syringae* pv. *tomato*		
DC3000	Wild type, Rif^r^	(Roine et al., 1997)
*P. fluorescens*		
EtHAn	*P. fluorescens* Pf0-1 carrying a working TTSS from *P. syringae* pv. *syringae*, Cm^r^	(Thomas et al., 2009)
*Agrobacterium tumefaciens*		
GV3101	Carries Vir plasmid encoding T-DNA transfer machinery, Rif^r^, Gm^r^	(Koncz and Schell, 1986)
*Saccharomyces cerevisiae*		
BF264-15Dau	*MAT*α* ade1 his2 leu2-3,112trp1-1a ura3*	Melissa G. Mitchum Lab
Plasmids		
pCR^™^8⁄GW⁄TOPO^®^	Gateway-compatible entry vector, Sp^r^	Invitrogen
pEDV6	Gateway-compatible version of pEDV3, Gm^r^	(Sohn et al., 2007)
pGBKT7-GW	Gateway-compatible version of pGBKT7, Km^r^	Melissa G. Mitchum Lab
pYEp51-*bax*	*bax* expression controlled by *GAL10* promoter, Amp^r^, LEU2	Melissa G. Mitchum Lab
pSITEII-3C1	Gateway-compatible binary vector for transiently over-expression of EGFP-fused protein *in planta*, Sp^r^	(Martin et al., 2009)

*Antibiotics were used at the following concentrations (μg/ml): Rifampicin (Rif) 100, Gentamycin (Gm) 50, Kanamycin (Km) 100, Spectinomycin (Sp) 50, Chloramphenicol (Cm) 30, and Ampicillin (Amp) 100.

### Plant Material

*P. vulgaris* plants (cultivar “Primel”) were grown in greenhouse chambers with 16-h light/8-h dark and 22°C/18°C.

*N. benthamiana* plants were grown in a growth room at an average temperature of 24°C (range, 20°C–26°C), with 45% to 65% relative humidity under long-day conditions (16 h light).

### Fungal Isolate, *in vitro* Structures of *U. appendiculatus*, and Inoculation of *P. vulgaris*

Urediospores of *U. appendiculatus* (SWBR1, laboratory collection, Phytopathology, University of Hohenheim, Stuttgart, Germany) were finely dispersed on polyethylene (PE) sheets using fine gauze material for sifting. The PE sheets were then sprayed with water using a chromatography vaporizer and afterward kept at 20°C, 95% humidity in the dark. For germ tubes (gt), the structures were harvested from the PE sheets after 4 h and for appressoria (ap) after 9 h. Also, for gt, smooth PE sheets were used, whereas for ap formation, the PE sheets were scratched using a brass brush. Formation of both gt and ap was checked microscopically. The structures were scraped from the PE sheets, dried by vacuum filtration, and stored in 2-ml tubes at −70°C after freezing in liquid nitrogen. For plant inoculation a watery suspension with 0.05% spores, 0.08% milk powder, and 0.01% Tween20 (w/v) was produced and stirred for 30 min at room temperature in the dark. The suspension was then sprayed on 21 days old *P. vulgaris* plants using a chromatography vaporizer. The inoculated plants were kept at 95% humidity in the dark at 15°C for 24 h before putting them back to greenhouse conditions with 16-h light/8-h dark and 15°C/13°C.

### RNA Preparation and cDNA Synthesis

*U. appendiculatus in vitro* structures were harvested as described above. Pieces from leaves of infected plants at different stages and uninfected plants were cut out with a cork borer (12 mm diameter, four pieces from different leaves per sample, roughly 100 mg), put into plastic tubes, frozen in liquid nitrogen, and kept at −70°C until RNA preparation. Frozen samples were homogenized using FastPrep^®^-24 MP (Biomedicals GmbH, Eschwege, Germany) at 4.5 m/s for three times 20 s with two 2-mm steel beads and with cooling the samples in liquid nitrogen between homogenization rounds. RNA purification was done using the Agilent Plant RNA Isolation Mini Kit (Agilent Technologies, Santa Clara, CA, USA) with adding an additional centrifugation step (2 min, 16,000 rcf, room temperature) between adding the extraction buffer to the homogenized tissue and using the mini prefiltration column. RNA concentration, quality, and integrity were checked measuring OD260/280 and running 1% denaturing agarose gels. Storage of the isolated RNA was at −70°C.

Before reverse transcription, RNA was treated with DNaseI (Thermo Fischer Scientific, Schwerte, Germany) using 1-µg RNA in a 10-µl reaction. For cDNA synthesis the RevertAid RT Reverse Transcription Kit (Thermo Fischer Scientific) was used. Twenty-microliter reactions starting with the DNaseI-treated RNA from above were performed following instructions from the manufacturer and using random hexamer primers. cDNA was stored at −20°C.

### Cloning

Inserts for cloning were produced by PCR on cDNA from urediospore RNA using nested PCR. First-round PCR was in 10-µl reactions with 1 µl 10× buffer, 1 µl 10 mM dNTP mix, 0.5 µl 0.1 µM f1 primers each (see **Table S1**), 1-µl cDNA, and 0.1 µl Long PCR Enzyme Mix (Thermo Fischer Scientific). Second round PCR was in 50-µl reactions with 5 µl 10× buffer, 5 µl 10 mM dNTP mix, 2.5 µl f2 primers each, 2 µl PCR product from the first round reaction, and 0.5 µl Long PCR Enzyme Mix. Cycling conditions were the same for both PCRs: initial denaturation 3 min 94°C, then 35 cycles with 20 s 95°C, 30 s 55°C, and 60 s 68°C; final elongation 7 min 68°C. Second round PCR products were purified using the peqGOLD Cycle-Pure Kit (PEQLAB Biotechnologie GmbH, Erlangen, Germany) and inserted into the pCR^™^8/GW/TOPO^®^ plasmid using the pCR^™^8/GW/TOPO TA Cloning^®^ Kit (Invitrogen, Life Sciences, Carlsbad, CA, USA), following the instructions of the manufacturer and using 4 µl of the purified PCR product. All constructs were verified by sequencing. For production of constructs without the signal peptide, the inserts were PCR amplified again using a third set of primers and again inserted into the pCR^™^8/GW/TOPO^®^ plasmid. All three sets of primers are supplied in **Table S1**.

### Bacterial Inoculation and Growth *in planta*

*N. benthamiana* plants used in this study were between 5 and 6 weeks old. All plant assays were performed by infiltrating a bacterial suspension into plant leaves with a needleless syringe. *Agrobacterium* strains were re-suspended in the induction buffer (100 μM acetosyringone, 10 mM MES, pH 5.6, and 10 mM MgCl_2_) and kept at room temperature for 3 h before infiltration, all other strains were re-suspended in inoculation buffer (10 mM MgCl_2_). The areas of bacterial infiltration were marked lightly with a Sharpie^®^ permanent marker. Levels of bacterial inoculum used in experiments are noted in the figures and legends.

### Agroinfiltration-Mediated Transient Expression in *N. benthamiana*

To create GFP-Uaca_N_ns_ constructs, the Uaca_N_ns_ were PCR amplified and cloned into the Gateway entry vector pCR^™^8⁄GW⁄TOPO^®^ (Invitrogen) and then recombined into the Gateway binary destination vector pSITEII-3C1 (**Table 1**). The resulting binary constructs were introduced into *A. tumefaciens* GV3101 using the freeze-thaw method (An et al., 1988). GFP was detected in agroinfiltrated *N. benthamiana* leaves using a Zeiss Axio Imager epi-fluorescence microscope at 48 h after infiltration.

### Yeast Cell Death Assays

Yeast cell death assays were performed following the procedure previously described (Laloux et al., 2010) with some modifications. Yeast strain BF264-15Dau carrying the plasmid Yep51-*bax*, which is inducible by galactose to express BAX protein, and Gateway-compatible pGBKT7-GW containing Uaca_N_ns_, was cultured in liquid SD/-Leu/-Trp media at 30°C for overnight before the yeast cells were resuspended in liquid YNB/Gal/-Leu/-Trp medium (Yeast Nitrogen Base 6.7 g/L, galactose 2%, -Leu/-Trp DO Supplement 0.64 g/L) for six-hour induction. Yeast cells were collected by centrifugation, washed in liquid YNB/Gal/-Leu/-Trp medium for at least four times, and adjusted to OD_600 nm_ = 1. The resulting yeast inoculum was serially diluted and placed on YNB/Gal/-Leu/-Trp plates. The plates were photographed at the fourth day after culturing at 30°C. Nematode effector 4F01, which consistently and strongly suppresses BAX cell death [Melissa G. Mitchum, personal communication and Qi et al. (2018)], and empty vector were included in each plate as positive and negative control, respectively.

### RT-qPCR Measurements

Real-time PCR reactions were run on a CFX96^™^ Real-Time-PCR System (Bio-Rad Laboratories, Hercules, CA, USA). The SensiFAST^™^ SYBR^®^ No-ROX mix (Bioline Reagents Ltd., London, UK) was used with primers at 0.4-µM final concentrations and 2-µl cDNA in 20-µl reactions. We used a two-step PCR protocol with an initial denaturation step of 95°C for 5 min, and then 5 s at 95°C, and 15 s at 60°C. Forty cycles were completed; then a melt curve analysis was run spanning 65 to 95°C with 0.5°C temperature increase per 5 s. Data logging and the determination of *C*q values were done using Bio-Rad CFX Manager 2.1 (Bio-Rad Laboratories). We used a threshold of 2,500 RFU, the estimated mean of thresholds set by automatic threshold determination. Means of the two technical replicates were calculated using the GenEx software package: GenEx 6.0.1.612 (MultiD Analyses AB, Göteborg, Sweden). Using GenEx, we also performed efficiency correction, normalization to the two reference genes, *Act* and *CytB* [determined as stable comparing several candidate reference genes; publication in preparation; strategy similar to the one described in Hirschburger et al. (2015) including all stages also used here] and calculated the expression relative to the gt sample. The geometric means of the three biological replicates were calculated in MS Excel.

We used Primer3 (Koressaar and Remm, 2007; Untergasser et al., 2012) for primer design and GeneRunner for additional checking and optimizing the primers. Primers were chosen to obtain amplicons between 150 and 250 bp in length. All primer pairs were tested for efficiency using dilution series with four tenfold dilutions prepared from cDNA of gt or 7 dpi stages. Primer data are summarized in **Table S2**.

## Results

### Pre-Selection of Candidate Effectors

Our study on the haustorial transcriptomes of *P. pachyrhizi* and *U. appendiculatus* resulted in a much higher number of predicted secreted proteins for *U. appendiculatus* (395) than for *P. pachyrhizi* (156) (Link et al., 2014). This prompted us to use a different cloning strategy. Instead of aiming at cloning all and starting with the largest, we focused on features that were in part already described in Link et al. (2014) to select among the predicted secreted proteins with the goal of cloning 40 to 50 secreted proteins. Faced with a similar problem, Petre et al. (2015) used the following criteria to select candidate effectors: haustorial expression, *in planta*-specific expression, no protein domain, Pucciniales specific, positive selection, induction during biotrophic growth, and homology to rust effectors. Due to the absence of genome sequence and time course transcriptome data, we chose specificity to Pucciniales as our primary criterion to select candidates. We preferentially selected candidates with homology to known rust effectors and excluded those with annotated functional domains. These criteria were applied to the protein families resulting from clustering analysis performed by Link et al. (2014) rather than to the single proteins. We then chose representative proteins from these families as candidates to be cloned based on whether they were the only *U. appendiculatus* protein in the family or the *U. appendiculatus* protein that fit the Hidden Markov model to the family best. We expected that this family-wise approach would provide us with a manageable number of candidates, and the ability to make general conclusions based on the protein representing each family. The *U. appendiculatus* candidates were termed Uaca_N, with the number N, indicating the order in which the candidates were selected and cloned.

For each Uaca_N, we first cloned the full ORF into the pCR^™^8 Gateway entry vector, and then we also produced a corresponding clone in pCR^™^8 minus the coding sequence for the predicted signal peptides, because our screens were dependent on intracellular expression or expression from the bacterial T3SS. The latter set of clones with no signal peptide were subsequently designated Uaca_N_ns_. The Uaca_N were cloned from cDNA derived from RNA that was extracted from *U. appendiculatus* SWBR1 urediospores. Because single-step PCR on this kind of cDNA proved unsatisfactory in earlier experiments, we used nested PCR for all Uaca_N. The resulting pCR^™^8 plasmids were tested for inserts by colony PCR and sequenced. The Uaca_N_ns_ were cloned from the pCR^™^8 plasmids containing the Uaca_N. All primers used for the cloning procedure are presented in **Table S1**. Only 31 of the 48 selected Uaca_N were successfully cloned and used in subsequent assays for cell death suppression in yeast and plants, PTI suppression, localization, and time course expression analysis. The description of the gene families and rationale for selection is provided in **Table 2** along with a summary of the results from each of the assays. Information, including the contig names that can be found in public databases, cloned ORF sequence, the protein sequence, protein length, cysteine content, and the predicted signal peptide that was removed is provided in **Data Sheet 1**.

**Table 2:** Effector candidates and results of the experimental assays. ΔHR, suppression of the hypersensitive response; ΔII, suppression of innate immunity; ΔBAX, suppression of yeast cell death. Subcellular localization and predicted subcellular localization (Pred): C, cytoplasm; N, nucleus; GE, gene expression; 0, no regulation, expression levels vary less than tenfold; − *in planta* down-regulation; + *in planta* up regulation (−/+ > tenfold, −−/++ > hundredfold, −−−/+++ > thousandfold; nt, not tested).

Name	Description of the gene families that the effector candidates belong to and where applicable the actual protein^a^	ΔHR	ΔII	ΔBAX	Subcellular localization	GE
Uaca_1 Ua_RTP1	Family of rust transferred proteins (Kemen et al., 2005; Puthoff et al., 2008). Likely functions are formation of fibrils (Kemen et al., 2013) and inhibition of proteases (Pretsch et al., 2013). Expressed *in planta* (cell wall fraction) [2].	−	−	Medium	All N, some C	nt
Uaca_2 Ua_RTP2	Family of rust transferred proteins (Kemen et al., 2005; Puthoff et al., 2008). Likely functions are formation of fibrils (Kemen et al., 2013) and inhibition of proteases (Pretsch et al., 2013). Expressed *in planta* (P30, cell membrane and organelle fraction) [2].	−	−	Medium	N+C	−
Uaca_3	Cluster 1_0_152 is mostly lineage specific for *U. appendiculatus*; also has a motif similar to RxLR [1].	−	−	Strong	N+C	0
Uaca_4	Cluster 398 [1]. Proteins in the cluster have 12 conserved cysteines, and some are highly expressed or up-regulated *in planta*. Uaca_4 itself is abundantly expressed and closest homolog to UfHSP42, highly expressed in the haustorium (Link and Voegele, 2008).	−	+	Strong	N+C	+
Uaca_5	Cluster 20 is specific to Pucciniales when only Basidiomycetes are concerned but contains members from other plant pathogens also. Horizontal gene transfer? [1]	−	+	Strong	C, aggregation	++
Uaca_7	Cluster 398 [1]. Proteins in the cluster have 12 conserved cysteines and some are highly expressed or up-regulated *in planta*. Uaca_7 itself is most abundantly expressed.	−	+	Medium	N+C	++
Uaca_9	Cluster 112 has most members [1] and contains *P. pachyrhizi *proteins (PpEC_23, de_novo_3939, de_novo_7164, de_novo_1784) (Qi et al., 2016; De Carvalho et al., 2017; Qi et al., 2018) showing defense suppression; of the Ua homologs Uaca_9 fits the HMM second best. Proteomics showed expression of the protein both during spore germination and *in planta* (mostly cytoplasmic fraction) [2].	+	+	Strong	N+C	0
Uaca_10	Cluster 112 has the most members [1] and contains *P. pachyrhizi* proteins (PpEC_23, de_novo_3939, de_novo_7164, de_novo_1784) (Qi et al., 2016; De Carvalho et al., 2017; Qi et al., 2018) showing defense suppression; of the Ua homologs Uaca_10 fits the HMM third best.	−	−	Medium	C, aggregation	0
Uaca_11	Cluster 2456, lineage specific to Pucciniaceae [1]. Fits the pattern best.	−	−	Strong	C	++
Uaca_12	Cluster 2456, lineage specific to Pucciniaceae [1]. Fits the pattern second best.	+	−	Medium	C	+++
Uaca_14	Cluster 1_2 is lineage specific to Pucciniales and has eight conserved cysteines and two additional motives [1]. Uaca_14 fits the pattern second best. Proteomics also found the protein expressed *in planta* in the cell wall fraction, as well as other proteins from the family: UAHYP_19A_R_H01, Ua_contig00019 [2].	+	−	Medium	C	++
Uaca_16	Cluster 2565 is lineage specific to Pucciniaceae, maybe even Uromyces. Uaca_16 is one of the few proteins with haustorial expression in the family. Proteins in the family have highly similar sequences, there are no cysteines, very high content of tyrosine (17 or 16%# or 27% wt).	−	−	Strong	C	−−−
Uaca_20	Cluster 145 has three conserved cysteines, and conserved aromatic residues.	−	−	Medium	N+C	+
Uaca_22	Cluster 874 has only few homologs per species, seven or three conserved cysteines plus one not conserved cysteine. Serine- and threonine-rich, also many aromatic aa.	+	−	Weak	C	0
Uaca_23	Cluster 2768 is lineage specific to Pucciniaceae, has six conserved cysteines and consists of very short proteins. Uaca_23 is the longest protein of the cluster. Proteomics found the protein both in germlings and *in planta* (cell wall fraction) [2].	−	−	Medium	C	0
Uaca_24	Cluster 1_0_162 appears specific to *U. appendiculatus*. It is a very small family, has very short proteins (100 aa) and four conserved cysteines.	−	−	Negative	C	++
Uaca_25	Cluster 1_63 is specific to rust fungi. Uaca_25 has the longest sequence of the family.	−	−	Negative	N+C	nt
Uaca_27	Cluster 2622 is specific to *U. appendiculatus*. It is a small family of short secreted proteins with cysteines.	−	−	Strong	N+C	nt
Uaca_28	Cluster 2917, basically just one gene with one homolog in *U. fabae*; specific to *Uromyces*.	−	+	Negative	N+C	−−
Uaca_31	Cluster 824 is not very conserved; N-terminus differs strongly but conserved cysteines are present. Lineage specific to Pucciniales.	−	−	Negative	N+C	nt
Uaca_32	Cluster 1293, only one homolog per species. By proteomics found *in planta*, cell wall fraction [2].	−	−	Strong	C	nt
Uaca_34	Cluster 2484 consists of only two very similar homologs. Very short proteins, two conserved cysteines. Lineage specific to Puccinales.	−	−	Strong	N+C	nt
Uaca_36	Cluster 1_229, only two homologs. Lineage specific for *U. appendiculatus*, 10 conserved cysteines	−	−	Strong	N+C	nt
Uaca_37	Cluster 464 is lineage specific to Pucciniales; eight conserved cysteines.	−	−	Negative	N+C	nt
Uaca_38	Cluster 1206 is lineage specific to Pucciniales; conserved cysteines.	−	−	Weak	N+C	nt
Uaca_40	Cluster 2240 is specific to Pucciniales, twelve conserved cysteines with a similar pattern to that of cluster 112.	−	−	Strong	N+C	nt
Uaca_41	Cluster 2826, specific to Pucciniaceae.	−	−	Medium	N+C	nt
Uaca_43	Cluster 3063 is lineage specific to *U. appendiculatus *and consists of only two very similar proteins with twelve or eighteen cysteines respectively, which might be splicing variants. Uaca_43 is the shorter protein.	−	−	Strong	C	nt
Uaca_44	Cluster 3112 is specific to Pucciniaceae; only one homolog per species	−	+	Weak	N+C	++
Uaca_45	Cluster 3113, specific to *U. appendiculatus*, six conserved cysteines, of the two proteins of the family Uaca_45 is slightly shorter and has more serines	−	−	Medium	N+C	nt
Uaca_46	Cluster 3121, lineage specific to Uromyces	−	−	Negative	C	nt

^a^Here we explain why each Uaca_N was chosen. Clustering and motif results refer to Link et al. (2014) [1]; proteomics results (Cooper et al., 2016) [2] are also mentioned. Additional literature has full citation in the table. No overlap between our candidates and silenced effector candidates (Cooper and Campbell, 2017) was found.

### Suppression of HR and Basal Defense by Uaca_Ns

**Figure 1:**
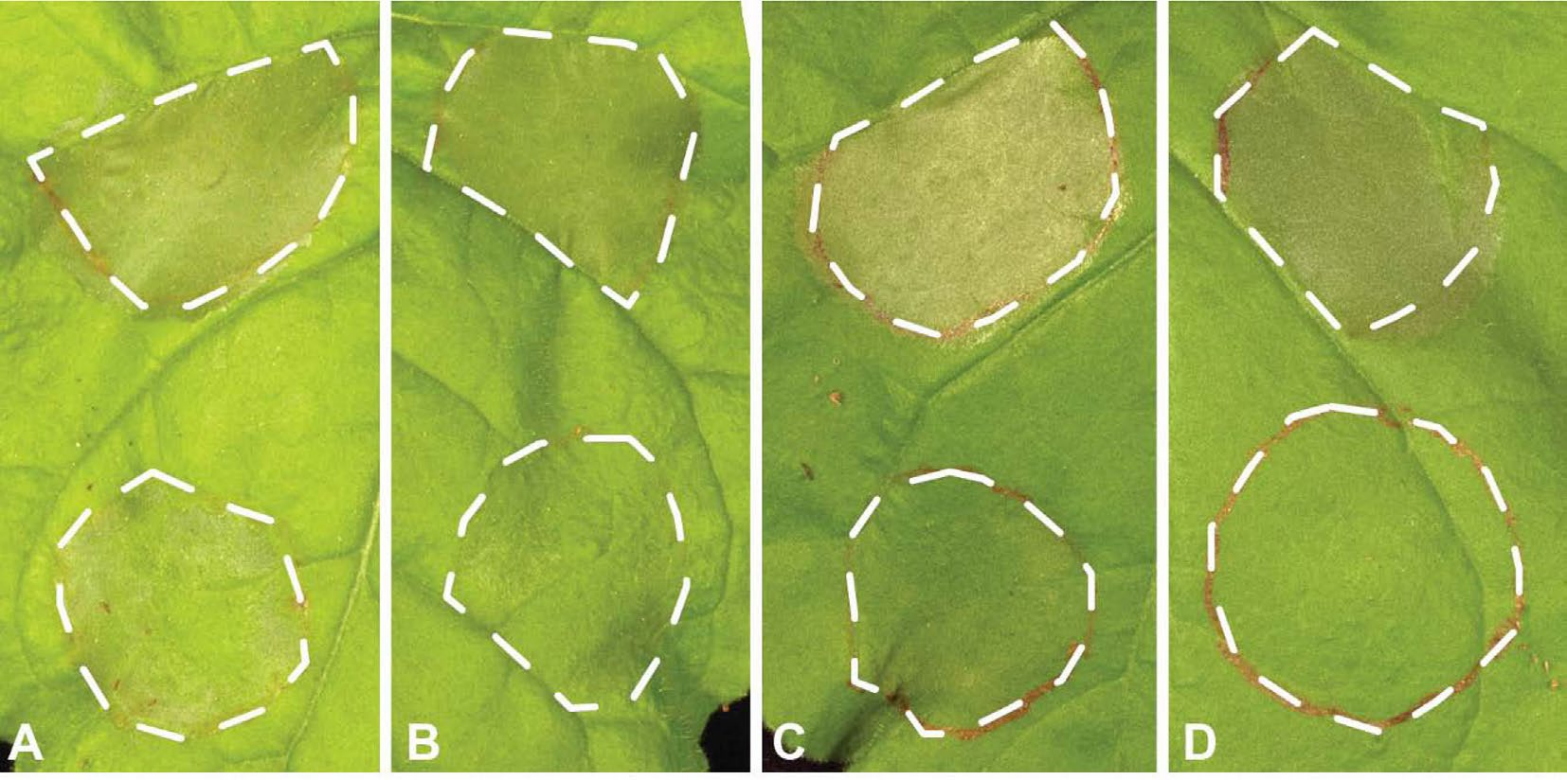
Four Uaca_Ns suppress HR induced by *Pst* DC3000 in *N. benthamiana*. The upper patch of each panel was infiltrated with *Pst* DC3000 with pEDV6 empty vector control, while the lower patch of each panel was infiltrated with *Pst* DC3000 expressing Uacas from pEDV6. **(A)** Uaca_9; **(B)** Uaca_12; **(C)** Uaca_14; **(D)** Uaca_22. The inoculum density was adjusted to OD_600nm_ = 0.02. Images were taken 48 hours-post-inoculation (hpi). Three independent repeats of this assay were performed for each Uaca.

The 31 Uaca_N_ns_ were transferred into the bacterial T3SS vector pEDV6 (effector detector vector 6), which previously has been used to deliver non-bacterial proteins into plants (Fabro et al., 2011; Upadhyaya et al., 2014; Qi et al., 2018). These 31 plasmids plus the empty pEDV6 were introduced into *Pst* DC3000 and *P. fluorescens* strain EtHAn (a non-pathogen engineered to carry the T3SS) (Thomas et al., 2009). *Pst* DC3000 induces HR in *N. benthamiana* due to recognition of the effector HopQ1-1 (Wei et al., 2007), which provides a straightforward system for screening Uaca_N_ns_ for the ability to suppress ETI when expressed from pEDV6 (Qi et al., 2016; Qi et al., 2018). To test for suppression of HR, *Pst* DC3000 strains carrying pEDV6 expressing the 31 Uaca_N_ns_ were blunt syringe-inoculated into leaves of *N. benthamiana*. Among the 31 strains, Uaca_9_ns_, Uaca_12_ns_, Uaca_14_ns_, and Uaca_22_ns_ were able to reproducibly suppress the HR caused by *Pst* DC3000 to different extents (**Figure 1**). The other 27 strains were able to cause HR in *N. benthamiana* leaves that was comparable to the pEDV6 empty vector control. These data demonstrate that four Uaca_N_ns_ can suppress ETI induced by HopQ1-1 in *N. benthamiana*.

**Figure 2:**
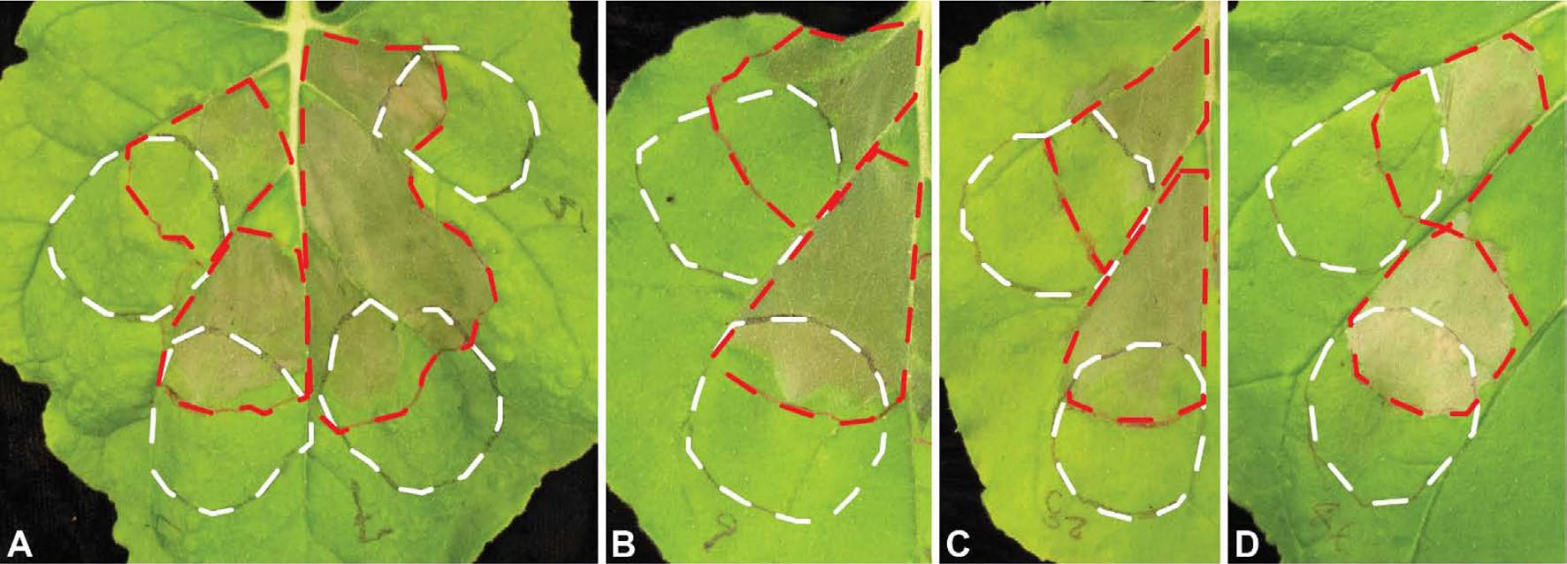
Suppression of basal defense by Uaca_Ns. The white dashed lines show an outline of the patches infiltrated with *P. fluorescens* strain EtHAn with empty pEDV6 vector control or expressing Uacas from pEDV6, and the red dashed lines show the patches infiltrated with *Pst* DC3000 seven hours later. **(A)** left upper patch, EtHAn with empty vector control; left lower patch, EtHAn with Uaca_4; right upper patch, EtHAn with Uaca_5; right lower patch, EtHAn with Uaca_7; **(B**, **C**, **D)** upper patch, EtHAn with empty vector control; lower patch, EtHAn with Uaca_9 **(B)**, Uaca_28 **(C)**, and Uaca_44 **(D)**. The inoculum density of EtHAn strains and *Pst* DC3000 strains was adjusted to OD_600nm_ = 0.2 and 0.02, respectively. Images were taken 48 hpi of EtHAn strains. At least three independent repeats of this assay were performed for each Uaca_N.

To test for suppression of basal defense, we used the assay established by Oh and Collmer (2005). The principle of this test is that non-pathogenic *P. fluorescens* EtHAn induces basal defense in *N. benthamiana*. *Pst* DC3000, on the other hand, triggers hypersensitive cell death. When *Pst* DC3000 is infiltrated into tissue that previously was infiltrated with EtHAn and, therefore, exhibits basal defense, no hypersensitive response is observed. The assay was successfully used to screen for *Phakopsora pachyrhizi* effectors (Qi et al., 2018). EtHAn strains carrying the 31 Uaca_N_ns_, together with empty vector control, were blunt syringe-inoculated into leaves of *N. benthamiana*, and 7 h later overlapping sites were inoculated with *Pst* DC3000. Of the 31 Uaca_N_ns_, six (Uaca_4, Uaca_5, Uaca_7, Uaca_9, Uaca_28, and Uaca_44) enabled *Pst* DC3000-induced HR, indicating that these six effector candidates are able to suppress the basal defense induced by EtHAn (**Figure 2**).

Protein size, number of cysteines, and ratio of cysteines have been implicated with the likelihood of proteins to have effector functions (Duplessis et al., 2012; Saunders et al., 2012; Sperschneider et al., 2018), and so we attempted to correlate these properties of the Uaca_Ns with the suppression of defenses. To find tendencies in these properties, we produced (pseudo) scatter blots with protein size as the X-axis and number of cysteines as the Y-axis (**Supplementary Figure 1**). Indeed, three of the four suppressors of HR and four of the six suppressors of basal defense are relatively rich in cysteine. This seems to indicate a tendency toward high cysteine content but no clear groupings could be seen in our plots.

### Suppression of BAX Induced Cell Death in Yeast by Uaca_Ns

**Figure 3:**
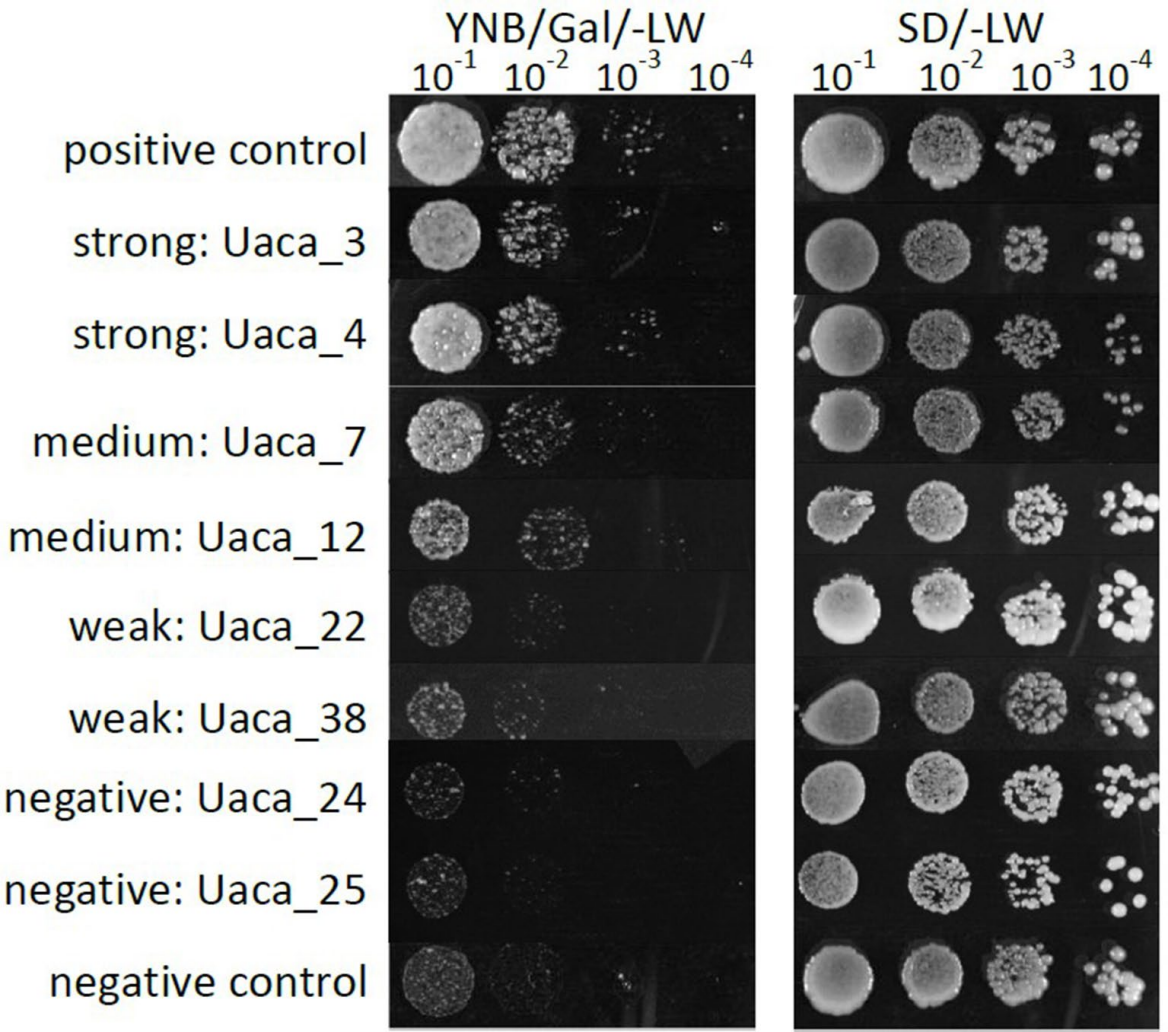
Examples of phenotypes of the yeast cell death suppression assay. Growth phenotypes of serial dilutions of yeast strains grown on medium that induced the expression of *bax*. Uaca_Ns with representative phenotypes were chosen for display. Strong suppression was defined by growth detected at the 10^−4^ dilution, Medium suppression was defined by growth detected at the 10^−3^ dilution, Weak suppression was defined by growth at the 10^−1^ or 10^−2^ dilution, and Non-suppression by no growth at any dilution. This assay was performed two independent times.

All 31 Uaca_N_ns_ were transferred to plasmid pGBKT7-GW, and the resulting constructs were transformed into yeast strain BF264-15Dau, which carries the plasmid YEp51-*bax* BF264-15Dau that confers *bax* gene expression under galactose induction (Zha et al., 1996). *Bax* induces cell death through effects like production of reactive oxygen species. We found that 25 of the 31 Uaca_Ns could suppress BAX cell death to various extents, and we categorized the suppression as strong, medium, weak, and negative (see **Figure 3** for representative examples). Into these categories fell 12, 10, 3, and 6 proteins, respectively. We also checked for tendencies using the same plots as for HR and PTI. Here, again, a tendency toward high cysteine content and also toward small protein size could be observed for the strong suppressors but no clear groups.

### Subcellular Localization of Uaca_Ns

**Figure 4:**
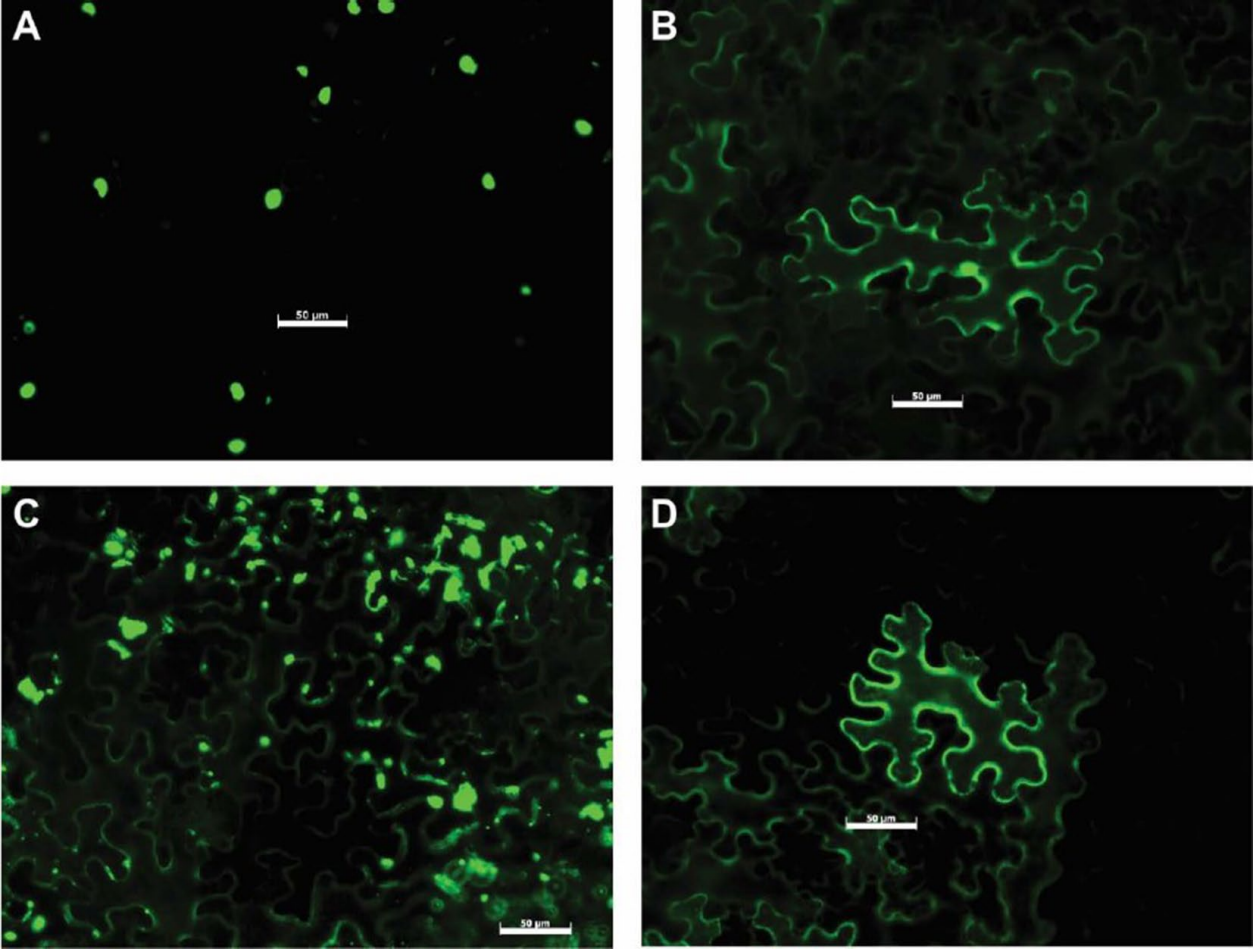
Representative examples of Uaca_N subcellular localization. **(A)** Uaca_1, localized in the nucleus; **(B)** Uaca_2, localized in both nucleus and cytoplasm; **(C)** Uaca_5, localized in the cytoplasm and forming aggregates; **(D)** Uaca_11, localized in the cytoplasm. The scale bar is equal to 50 µm. Images were taken 48 hpi. Two replications of the imaging were performed, and at least four representative images were taken each time.

To determine the subcellular localization of Uaca_Ns *in planta*, 31 Uaca_N_ns_ were cloned into pSITEII-3C1 (Martin et al., 2009) to fuse the GFP reporter to their amino termini. The GFP-Uaca_N_ns_ fusion proteins were transiently expressed in *N. benthamiana* by agroinfiltration. GFP fluorescence was visualized by epi-fluorescence microscopy in *N. benthamiana* epidermal cells. There were three basic patterns of GFP distribution in the cells: cytoplasmic, nuclear, and cytoplasmic + nuclear (**Figure 4**, **Supplementary Figure 2**, and **Table 2**). Of the 31 GFP-Uaca_N_ns_ fusion proteins, 18 were distributed in both the nucleus and the cytoplasm (e.g. **Figure 4B**). Another 12 were observed only in the cytoplasm and excluded from the nucleus (e.g. **Figure 4D**), 2 of which appear to be located mainly in small bodies, which could be organelles, other subcellular compartments, or artifacts of protein aggregation (**Figure 4C**). Only one, GFP-Uaca_1, was mostly located in the nucleus (**Figure 4A**). These findings indicate that *U. appendiculatus*, similar to other rust fungi, may deliver effector proteins to a variety of distinct host cell compartments (Petre et al., 2015).

### Expression Patterns of Uaca_Ns

**Figure 5:**
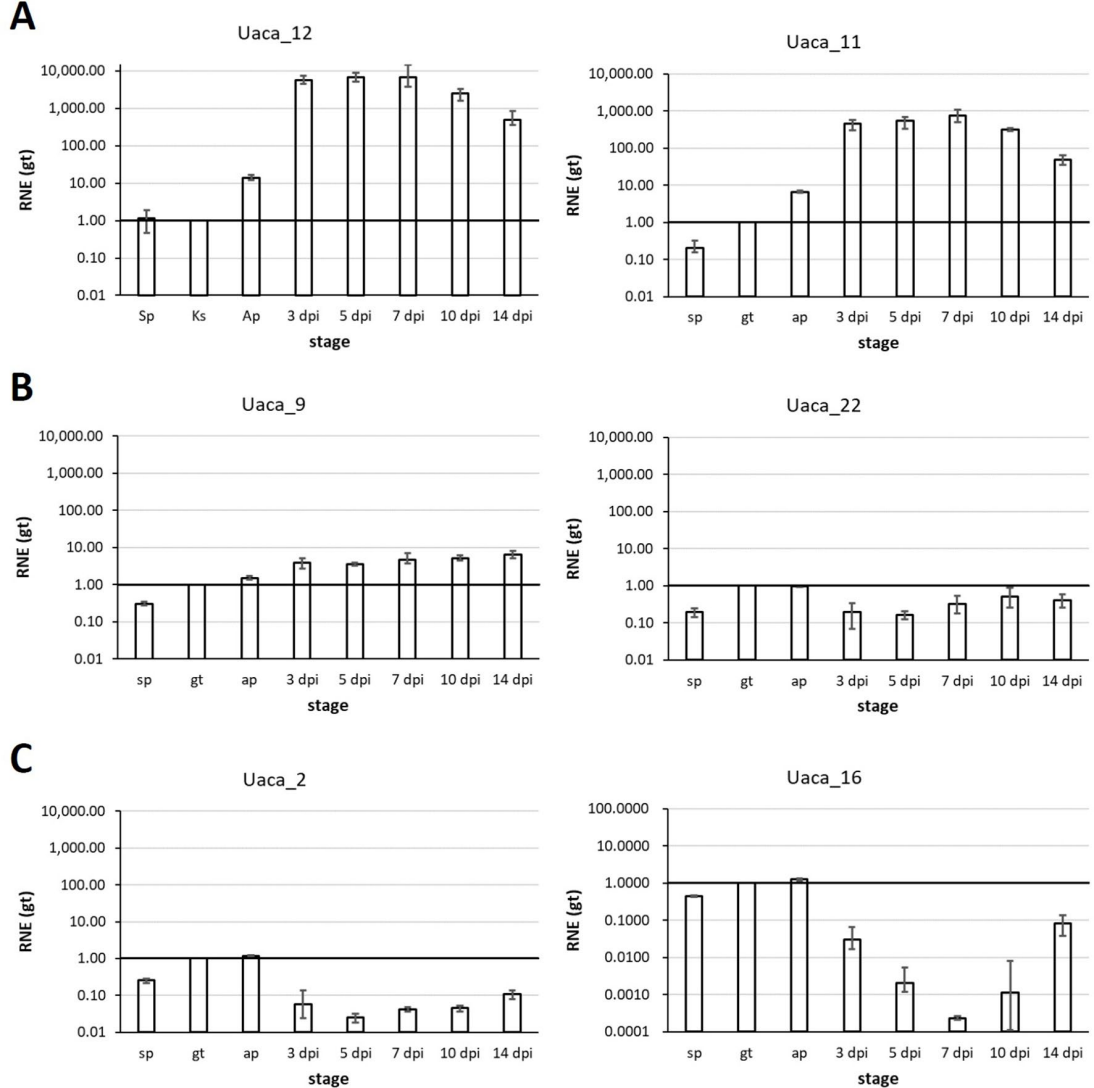
Representative mRNA expression patterns of Uaca_Ns. Stages tested: *in vitro*: ungerminated urediospore (sp), germ tube (gt), appressorium (ap); *in planta*: 3, 5, 7, 10, and 14 days post inoculation (dpi). **(A)** Examples of Uaca_Ns that were strongly up-regulated *in planta*; **(B)** examples of Uaca_Ns with little or no change in expression across the stages; **(C)** examples of Uaca_Ns that were down-regulated *in planta*. Columns show the geometric means of three biological replicates; error bars indicate maximum and minimum values. All values are relative to the gt stage; RNE, relative normalized expression.

For 17 Uaca_Ns that were assumed to have effector function after the assays described above, we also tested the expression pattern (**Figure 5**, **Supplementary Figure 3**). Using RT-qPCR, we tested Uaca_N expression in three stages produced *in vitro* (ungerminated urediospore, germ tube, and appressorial stage) and five *in planta* stages (3, 5, 7, 10, and 14 dpi). The first three *in planta* stages are meant to represent infection and haustoria formation, and the latter two time points encompass sporulation and senescence. *In planta* stages earlier than 3 dpi were not tested because of the low proportion of fungal RNA in total RNA prepared from inoculated leaves, which causes highly variable results. Based on the fact that the sequences were initially found in the haustorial transcriptome, we expected the genes to be up-regulated *in planta*. This was the case for nine of the 17 effector candidates tested for gene expression. Two Uaca_Ns were up-regulated >10-fold (Uaca_4, Uaca_20), six >100fold (Uaca_5, Uaca_7, Uaca_11, Uaca_14, Uaca_24, Uaca_44), and one >1000-fold (Uaca_12) (**Figure 5A**, **Supplementary Figure 3**). On the other hand five genes showed no or little differential expression, including Uaca_9 and Uaca_10 (**Figure 5B**, **Supplementary Figure 3**). Three of the tested Uaca_Ns, including Ua_RTP2, were even down-regulated *in planta*, Ua_RTP2 >10fold, the others >100fold. All three genes showed maximal expression in the appressorial stage (**Figure 5C**, **Supplementary Figure 3**). The high expression of some candidate effectors at the appressorial stage suggests that they have functions that are needed during early infection processes.

## Discussion

Relatively little is known about the functions of effectors produced by rust fungi. There are very few examples of *bona fide* effectors known for the rust fungi (Petre et al., 2014). These few were either identified as avirulence (Avr) proteins (Ellis et al., 2007; Upadhyaya et al., 2014) or directly localized inside plant cells by immunofluorescence microscopy (Kemen et al., 2005). Some of these effectors have been analyzed down to the crystal structure (Wang et al., 2007; Ve et al., 2013) and functions were inferred (Wan et al., 2019) but even for most of those effectors, little is known about their biochemical functions and their roles in virulence (Petre et al., 2014). In contrast to this, it is hypothesized that there are many effectors and that they have important functions, especially in suppression of host resistance. This hypothesis is supported by results with other plant pathogens, such as Oomycetes like *Phytophthora infestans*, where hundreds of effectors were found (Tyler et al., 2006). Results from genome or transcriptome sequencing of different rust species are consistent with oomycete data in that large numbers of secreted proteins have been predicted, and most of these are of unknown function, opening the possibility that they could have effector function (Duplessis et al., 2011; Cantu et al., 2013; Nemri et al., 2014).

In comparison to bacteria and oomycetes, research on rust effectors is hampered by the fact that so far no common sequence feature for effectors has been identified. To overcome this limitation, other criteria were used to determine whether secreted proteins are likely effectors. Saunders et al. (2012) for example built an effector prediction pipeline based on the following criteria: expression induced *in planta*, similarity to HESPs or Avrs, known effector motif or NLS, small and cysteine-rich, repeat containing, long intergenic region, or no PFAM domain. The central element of this pipeline is clustering of the proteins into families. In our study on the haustorial transcriptomes of *P. pachyrhizi* and *U. appendiculatus* (Link et al., 2014), we also used clustering to identify families of secreted proteins, and then established whether the family was specific to the rusts or lineages within the rusts, which was a paramount criterion for classifying a secreted protein as an effector candidate.

The other approaches to identify effectors are experimental screens. Secreted proteins can be screened against cultivars with known resistance genes to find Avrs, which yielded an effector in coffee rust (Maia et al., 2017), or against non-host plants to identify effector-specific recognition (Dagvadorj et al., 2017). Very recently, a screen for effectors suppressing RNA silencing was successful (Yin et al., 2019). Most important are screens based on subcellular localization (Petre et al., 2015; Petre et al., 2016) or suppression of defense responses (Sohn et al., 2007). It has been discussed that the protein fusion has an effect on the outcome of such assays, and that results could differ between experiments with N-terminal fusions and C-terminal fusions. We recently implemented a combination of different screens to identify secreted proteins of *P. pachyrhizi* with effector-like functions (Qi et al., 2018). Here we used N-terminal fusions based on the rationale that the tags are replacing the signal peptide. For the first effector candidate that we identified this way, PpEC23 (Qi et al., 2016), we found that the C-terminus is important for its interactions with a host-transcription factor. This finding reassured us that N-terminal fusions can be functional. We cannot exclude that for some effector candidates C-terminal fusions would be the better alternative but it was not feasible for us to do the screens with both N- and C-terminal fusions.

For *U. appendiculatus*, we combined our theoretical predictions with our experimental screens. The selective criteria we used to identify Uaca_Ns yielded a higher proportion of secreted proteins with positive results in the *in planta* assays than we previously observed with the *P. pachyrhizi* candidate effectors. Six effector candidates or 19% could suppress basal defense compared to 14%. With only six negatives, the vast majority of our candidates could suppress BAX-induced cell death in yeast. Most striking was that we could find four Uaca_Ns that can suppress the hypersensitive response whereas only one such protein could be found for *P. pachyrhizi*. Therefore, we conclude that a pre-selection of candidate effectors based on sequence features can help reduce experimental efforts.

Also, by covering many different protein families our results can point to other proteins, which may be valuable targets for testing. Because many of the families of effector candidates extend between species of the rust fungi, researchers interested in different species may profit from our results. As a caution, it should be mentioned that it is highly likely that expression patterns, targets, and in consequence, functions of effectors in the same family are different. Therefore, the results, even though they do not constitute functional characterization, should not be directly transferred to other proteins of the family—rather, our results are meant to point at interesting gene families. Several studies have used clustering to group families of secreted proteins, but it is challenging to make comparisons among the families from these different studies. So far, matching our gene families to the clusters found by other groups, e.g. Saunders et al. (2012), Cantu et al. (2013), Nemri et al. (2014), or De Carvalho et al. (2017) remains a laborious exercise. A second challenge is that transcriptome studies are inherently incomplete. We expect that as more genomes become available more comprehensive comparisons can be performed. This is an especially exciting potential outcome of the rust pangenomics project, “Reference Genomes for 50 Rust Fungi” (https://jgi.doe.gov/csp-2018-duplessis-reference-genomes-50-rust-fungi/).

A few genes and gene families that merit special attention are discussed below.

The first two candidates were chosen to obtain further information on proteins of the RTP family (Puthoff et al., 2008; Pretsch et al., 2013). *Uf*-RTP1p has been shown to induce fibril formation inside host cells, and it also has protease inhibitor activity (Kemen et al., 2013; Pretsch et al., 2013). These two functions indicate a possible structural role for *Uf*-RTP1p as well as a role in protecting the fungus against an active defense response mediated by proteases. Here, we found that both *Ua*_RTP1 (Uaca_1) and *Ua*_RTP2 (Uaca_2) did not suppress plant defenses in the assays performed. Therefore, no additional functions can be added to the RTP portfolio. At this time, we are not sure if the suppression of BAX induced cell death that was observed (medium for both Ua_RTP1 and Ua_RTP2) has relevance. The localization of our GFP fusions nicely coincide with the immunolocalization of the native protein *Uf*_RTP1p and also *Us*_RTP1p (Kemen et al., 2005). Kemen et al. (2005) could observe that *Uf*_RTP1p first accumulates around the haustorium or more specifically in the extrahaustorial matrix (Kemen et al., 2013). In cells infected with older haustoria, the protein was found in the cytoplasm and also in the nucleus. Also, an NLS was found in the *Uf*_RTP1 sequence that could account for the nuclear localization. The GFP fusion proteins are also localized in the cytoplasm and the nucleus inside *N. benthamiana* cells. This coincidence indicates that heterologous expression of GFP fusions of effector candidates in *N. benthamiana* can correctly predict the targeting of the native effector protein. On the other hand, the study of Cooper et al. (2016) found Ua_RTP1 preferentially in cell walls. The latter proteomic result can be explained by the finding that *Uf*-RTP1p first accumulates in the extrahaustorial matrix before also being imported into the plant cytoplasm. The result for gene expression for Uaca_2 was surprising. The gene is most highly expressed in the appressorial stage but down-regulated *in planta*. This seems to be in contradiction to the results from Cooper et al. (2016). This discrepancy could be explained by the different experimental approaches targeting proteins and transcripts respectively, however. Also, the fungal isolates and the plant genotypes used in the respective experiments were different. Our results here indicate that proteins from the RTP family may have different expression patterns and since they end up in different fungal structures also may have different functions.

Uaca_9 and Uaca_10 were chosen as members of cluster 112, which is lineage specific to Pucciniales. The predominant feature of members of this cluster is a motif of 10 conserved cysteines. *Pp*EC_23, the first effector candidate from *P. pachyrhizi* for which we could show suppression of plant immunity (Qi et al., 2016; Qi et al., 2018) belongs to this cluster. Three more *P. pachyrhizi* proteins from this family were also tested (De Carvalho et al., 2017), and these also could suppress immunity in *N. benthamiana*. All the GFP fusion proteins were localized in the cytoplasm and nucleus with *Pp*EC_23 exhibiting strong aggregation. It is intriguing that of the two *U. appendiculatus* proteins tested here, Uaca_9 showed clear suppression of plant immunity and also strong suppression of BAX-induced cell death, whereas Uaca_10 did not suppress either basal defense or the hypersensitive response. The localization of both proteins was consistent with other members of the family from *P. pachyrhizi* with Uaca_10 also aggregating. The mRNA expression of Uaca_9 was induced by less than 10-fold *in planta* and Uaca_10 mRNA transcripts did not significantly change. The lack of dynamic changes in the expression of these two Uaca_Ns is consistent with *Pp*EC_23, which had the strongest gene expression in the appressorial stage. These observations suggest that the effectors from this gene family may be needed throughout the infection process. Overall our results provide further evidence that the gene family represented by cluster 112 is a highly interesting family of effector candidates in *U. appendiculatus* as well as in *P. pachyrhizi*. In particular, the domain structure of these proteins and how the domains contribute to defense suppression through interactions with host proteins is of interest.

Seven more Uaca_Ns stand out as highly likely effectors, especially because they either suppress HR or PTI. Since we based the choice of effectors on interesting features of the corresponding protein families, these are highlighted here. To our knowledge, no other proteins from these families were experimentally validated so far. Uaca_4 and Uaca_7 belong to cluster 398, a cluster made up of proteins highly expressed or up-regulated in the haustorium (Link et al., 2014). These are small, cysteine-rich proteins with a pattern of 12 cysteines. Cluster 398 corresponds to tribe 110, which was identified by Saunders et al. (2012). Our RT-qPCR results showed that Uaca_4 and Uaca_7 are also strongly up-regulated *in planta* by 3 dpi and, therefore, are expressed in a manner consistent with previous results for this family. Both proteins were able to suppress BAX-induced cell death in yeast and innate immunity, and they were localized to the cytoplasm and the nucleus when expressed in *N. benthamiana*. Most importantly, these Uaca_Ns suppressed PTI in the overlapping infiltration assay (**Figure 2**). These data coupled with the small, cysteine-rich nature of these proteins provides strong indicators that members of cluster 398 should be investigated further to understand the mechanisms by which they function in rust–host interactions. Uaca_5 belongs to cluster 20 (Saunders et al. (2012): tribe76) for which our phylogenetic analysis indicates that it could have been acquired by the Pucciniales by horizontal gene transfer relatively early, before most of the Pucciniales species were separated (Link et al., 2014). Another explanation for this phenomenon might be that all other (non biotrophic) basidiomycete species lost the gene because it is only useful in pathogenesis. All our data support the assumption that this protein is an effector: it suppresses innate immunity and yeast cell death, and it is strongly up-regulated *in planta*. Uaca_12 and Uaca_11 belong to cluster 2465 (Saunders et al. (2012): tribe213). Although both proteins are strongly up-regulated *in planta* and suppress yeast cell death, Uaca_12 can also suppress HR, which makes it a highly interesting effector candidate. Uaca_14 also suppresses the HR as well as yeast cell death. Cluster 1_2 that it belongs to (Saunders et al. (2012): tribe38), has conserved cysteines. The expression pattern also fits to what is expected for effectors. Finally, Uaca_22 belongs to cluster 874 that has members in several rust species but only few homologs per species. There is suppression of the HR but no strong regulation of the gene.

Our study provides experimental evidence for effector-like functions of proteins from several families of *U. appendiculatus* effector candidates. The data presented here lays a strong foundation for further work to characterize *U. appendiculatus* effectors, which may lead to identification of effector targets and novel sources of resistance in common bean. In addition, our study also led to some general findings or conclusions. To begin with, it became clear that selecting candidate effectors based on Pucciniales specificity of the gene family was useful as noted from the higher ratio of positive results in our *U. appendiculatus* screens versus previous screens of *P. pachyrhizi* effector candidates (Qi et al., 2018). EffectorP (Sperschneider et al., 2016; Sperschneider et al., 2018) that is trained to predict effectors from sequence features of proteins secreted by pathogenic fungi was not yet available when we did our candidate selection. Recently, we have run the predictions and found that EffectorP1.0 predicted 16 of our candidates as effectors, EffectorP2.0 15 (**Data Sheet 1**). The predictions differed between the two algorithms for three effector candidates. Among the candidates predicted as effectors are both *Ua*_RTPs and also the homologs of PpEC23, which is not surprising since both *Uf*_RTP1 and PpEC23 belong to the positive training set for EffectorP2.0. In this case the prediction of EffectorP is exactly the same as with our approach. A true test of the algorithms is not possible with our data, mostly because our results do not yet identify *bona fide* effectors. In our opinion, EffectorP would have been useful in conjunction with lineage specificity and gene family when selecting effector candidates for this study.

We also identified slight trends regarding size and cysteine content of the effector candidates in suppression of yeast cell death and plant defenses. Our RT-qPCR measurements of gene expression indicate that effector expression can be highly diverse even though all effector candidates in this study were originally identified as being expressed in haustoria. Three candidates, for which there is strong evidence that they are effectors, showed relatively small changes in mRNA expression or they were even down-regulated *in planta*. These results indicate that up-regulation *in planta* may not pose as strong evidence for effector function as previously thought, or that mechanisms of posttranscriptional regulation should also be considered as an important mechanism for controlling effector expression. On the other end of our candidate spectrum, we also found one gene (Uaca_24) that was highly up-regulated *in planta*, but it was not able to suppress immune responses in the assays performed. This could be an effector with a different function, such as inducing the release of nutrients to the pathogen or other changes in infected cells or tissues. Additional assays for defense suppression and nutrient release are needed to further explore the functions of this and other candidate effectors.

## Data Availability Statement

All datasets generated for this study are included in the manuscript/**Supplementary Files**.

## Author Contributions

MQ, RV, SW, and TL planned and designed the study; TL chose and cloned the candidate genes; YM recloned Uaca coding sequences into vectors for use in all the expression assays; MQ and LD performed assays for PTI and ETI suppression; JS performed bax cell death suppression experiments, JG performed the epi-fluorescence microscopy, MR did the RT-qPCR measurements; MQ, SW, and TL wrote the manuscript. All authors read and approved the submitted manuscript version.

## Funding

This work was supported by the NSF IOS (award 1551452 to SW), USDA NIFA Hatch project 3808, and State of Iowa funds. LD was supported by a fellowship from The National Council for Scientific and Technological Development (CNPq), Brazil. RV, TL, and JS were supported by a PPP program of the DAAD (PPP 57130080).

## Conflict of Interest

The authors declare that the research was conducted in the absence of any commercial or financial relationships that could be construed as a potential conflict of interest.

## Supplementary Material

Data Sheet 1 (.xlsx) Microsoft excel file containing sequence IDs that can be found in the public databases (alternative name), the open reading frame sequences together with the peptide sequences and information about the predicted signal peptide that was removed in cloning. The length of the full peptides is also included as well as the number of cysteines contained in the proteins (no. C) together with the percentage of cysteines (% C). Prediction results of EffectorP1 and EffectorP2 are incorporated in this table to complement information on effector candidate prediction. EffectorP1 and EffectorP2 were not available when this study was initiated, so their output could not be used for candidate effector selection criteria.

## References

[B1] An G. Ebert P. R. Mitra A. Ha S. B. (1988). “Binary vectors,” in Plant Molecular Biology Manual. (Dordrecht: Kluwer Academic Publishers), 1–19. 10.1007/978-94-017-5294-7_3

[B2] Cantu D. Segovia V. Maclean D. Bayles R. Chen X. Kamoun S. (2013). Genome analyses of the wheat yellow (stripe) rust pathogen *Puccinia striiformis* f. sp. *tritici* reveal polymorphic and haustorial expressed secreted proteins as candidate effectors. BMC Genomics 14, 270. 10.1186/1471-2164-14-270 23607900 PMC3640902

[B3] Cooper B. Campbell K. B. (2017). Protection against common bean rust conferred by a gene-silencing method. Phytopathology 107, 920–927. 10.1094/PHYTO-03-17-0095-R 28437139

[B4] Cooper B. Campbell K. B. Beard H. S. Garrett W. M. Islam N. (2016). Putative rust fungal effector proteins in infected bean and soybean leaves. Phytopathology 106, 491–499. 10.1094/PHYTO-11-15-0310-R 26780434

[B5] Dagvadorj B. Ozketen A. C. Andac A. Duggan C. Bozkurt T. O. Akkaya M. S. (2017). A *Puccinia striiformis* f. sp. *tritici* secreted protein activates plant immunity at the cell surface. Sci. Rep. 7, 1141. 10.1038/s41598-017-01100-z 28442716 PMC5430700

[B6] De Carvalho M. C. Costa Nascimento L. Darben L. M. Polizel-Podanosqui A. M. Lopes-Caitar V. S. Qi M. (2017). Prediction of the *in planta Phakopsora pachyrhizi* secretome and potential effector families. Mol. Plant Pathol. 18, 363–377. 10.1111/mpp.12405 27010366 PMC6638266

[B7] Duplessis S. Cuomo C. A. Lin Y. C. Aerts A. Tisserant E. Veneault- Fourrey C. (2011). Obligate biotrophy features unraveled by the genomic analysis of rust fungi. Proc. Natl. Acad. Sci. U.S.A. 108, 9166–9171. 10.1073/pnas.1019315108 21536894 PMC3107277

[B8] Duplessis S. Joly D. L. Dodds P. N. (2012). “Rust effectors,” in Effectors in plant-microbe interactions. Eds. Martin F. Kamoun S. (Chichester: John Wiley & Sons), 155–194. 10.1002/9781119949138.ch7

[B9] Ellis J. G. Dodds P. N. Lawrence G. J. (2007). Flax rust resistance gene specificity is based on direct resistance–avirulence protein interactions. Annu. Rev. Phytopathol. 45, 289–306. 10.1146/annurev.phyto.45.062806.094331 17430087

[B10] Fabro G. Steinbrenner J. Coates M. Ishaque N. Baxter L. Studholme D. J. (2011). Multiple candidate effectors from the oomycete pathogen *Hyaloperonospora arabidopsidis* suppress host plant immunity. PLoS Pathogens 7, e1002348. 10.1371/journal.ppat.1002348 22072967 PMC3207932

[B11] Hirschburger D. Müller M. Voegele R. Link T. (2015). Reference genes in the pathosystem *Phakopsora pachyrhizi*/soybean suitable for normalization in transcript profiling. Int. J. Mol. Sci. 16, 23057–23075. 10.3390/ijms160923057 26404265 PMC4613351

[B12] Kemen E. Kemen A. Ehlers A. Voegele R. Mendgen K. (2013). A novel structural effector from rust fungi is capable of fibril formation. Plant J. 75, 767–780. 10.1111/tpj.12237 23663217

[B13] Kemen E. Kemen A. C. Rafiqi M. Hempel U. Mendgen K. Hahn M. (2005). Identification of a protein from rust fungi transferred from haustoria into infected plant cells. Mol. Plant-Microbe Interact. 18, 1130–1139. 10.1094/MPMI-18-1130 16353548

[B14] Koncz C. Schell J. (1986). The promoter of T_L_-DNA gene *5* controls the tissue-specific expression of chimaeric genes carried by a novel type of *Agrobacterium* binary vector. Mol. Gen. Genet. 204, 383–396. 10.1007/BF00331014

[B15] Koressaar T. Remm M. (2007). Enhancements and modifications of primer design program Primer3. Bioinformatics 23, 1289–1291. 10.1093/bioinformatics/btm091 17379693

[B16] Laloux G. Deghelt M. De Barsy M. Letesson J. J. De Bolle X. (2010). Identification of the essential *Brucella melitensis* porin Omp2b as a suppressor of Bax-induced cell death in yeast in a genome-wide screening. PLoS One 5, e13274. 10.1371/journal.pone.0013274 20949000 PMC2952587

[B17] Lawrence G. J. Dodds P. N. Ellis J. G. (2010). Transformation of the flax rust fungus, *Melampsora lini*: selection *via* silencing of an avirulence gene. Plant J. 61, 364–369. 10.1111/j.1365-313X.2009.04052.x 19874543 PMC3142615

[B18] Liebenberg M. M. Pretorius Z. A. (2010). Common bean rust: pathology and control. Hoboken, New Jersey. 10.1002/9780470543672.ch1

[B19] Link T. I. Lang P. Scheffler B. E. Duke M. V. Graham M. A. Cooper B. (2014). The haustorial transcriptomes of *Uromyces appendiculatus* and *Phakopsora pachyrhizi* and their candidate effector families. Mol. Plant Pathol. 15, 379–393. 10.1111/mpp.12099 24341524 PMC6638672

[B20] Link T. I. Voegele R. T. (2008). Secreted proteins of *Uromyces fabae*: similarities and stage specificity. Mol. Plant Pathol. 9, 59–66. 10.1111/j.1364-3703.2007.00448.x 18705884 PMC6640452

[B21] Maia T. Badel J. L. Marin-Ramirez G. Rocha C. M. Fernandes M. B. Da Silva J. C. (2017). The *Hemileia vastatrix* effector HvEC-016 suppresses bacterial blight symptoms in coffee genotypes with the SH1 rust resistance gene. New Phytol. 213, 1315–1329. 10.1111/nph.14334 27918080 PMC6079635

[B22] Martin K. Kopperud K. Chakrabarty R. Banerjee R. Brooks R. Goodin M. M. (2009). Transient expression in *Nicotiana benthamiana* fluorescent marker lines provides enhanced definition of protein localization, movement and interactions *in planta* . Plant J. 59, 150–162. 10.1111/j.1365-313X.2009.03850.x 19309457

[B23] Nemri A. Saunders D. G. Anderson C. Upadhyaya N. M. Win J. Lawrence G. J. (2014). The genome sequence and effector complement of the flax rust pathogen *Melampsora lini* . Front. Plant Sci. 5, 98. 10.3389/fpls.2014.00098 24715894 PMC3970004

[B24] Oh H. S. Collmer A. (2005). Basal resistance against bacteria in *Nicotiana benthamiana* leaves is accompanied by reduced vascular staining and suppressed by multiple *Pseudomonas syringae* type III secretion system effector proteins. Plant J. 44, 348–359. 10.1111/j.1365-313X.2005.02529.x 16212612

[B25] Petre B. Joly D. L. Duplessis S. (2014). Effector proteins of rust fungi. Front. Plant Sci. 5, 416. 10.3389/fpls.2014.00416 25191335 PMC4139122

[B26] Petre B. Saunders D. G. Sklenar J. Lorrain C. Krasileva K. V. Win J. (2016). Heterologous expression screens in *Nicotiana benthamiana* identify a candidate effector of the Wheat Yellow Rust pathogen that associates with processing bodies. PLoS One 11, e0149035. 10.1371/journal.pone.0149035 26863009 PMC4749346

[B27] Petre B. Saunders D. G. Sklenar J. Lorrain C. Win J. Duplessis S. (2015). Candidate effector proteins of the rust pathogen *Melampsora larici-populina* target diverse plant cell compartments. Mol. Plant-Microbe Interact. 28, 689–700. 10.1094/MPMI-01-15-0003-R 25650830

[B28] Pretsch K. Kemen A. C. Kemen E. Geiger M. Mendgen K. Voegele R. T. (2013). The rust transferred proteins - a new family of effector proteins exhibiting protease inhibitor function. Mol. Plant Pathol. 14, 96–107. 10.1111/j.1364-3703.2012.00832.x 22998218 PMC6638633

[B29] Puthoff D. P. Neelam A. Ehrenfried M. L. Scheffler B. E. Ballard L. Song Q. (2008). Analysis of expressed sequence tags from *Uromyces appendiculatus* hyphae and haustoria and their comparison to sequences from other rust fungi. Phytopathology 98, 1126–1135. 10.1094/PHYTO-98-10-1126 18943459

[B30] Qi M. Grayczyk J. P. Seitz J. M. Lee Y. Link T. I. Choi D. (2018). Suppression or activation of immune responses by predicted secreted proteins of the soybean rust pathogen *Phakopsora pachyrhizi* . Mol. Plant-Microbe Interact. 31, 163–174. 10.1094/MPMI-07-17-0173-FI 29144203

[B31] Qi M. Link T. I. Müller M. Hirschburger D. Pudake R. N. Pedley K. F. (2016). A small cysteine-rich protein from the Asian Soybean Rust fungus, *Phakopsora pachyrhizi*, suppresses plant immunity. PLoS Pathogens 12, e1005827. 10.1371/journal.ppat.1005827 27676173 PMC5038961

[B32] Roine E. Wei W. Yuan J. Nurmiaho-Lassila E.-L. Kalkkinen N. Romantschuk M. (1997). Hrp pilus: an *hrp*-dependent bacterial surface appendage produced by *Pseudomonas syringae* pv. *tomato* DC3000. Proc. Natl. Acad. Sci. U.S.A. 94, 3459–3464. 10.1073/pnas.94.7.3459 9096416 PMC20392

[B33] Saunders D. G. Win J. Cano L. M. Szabo L. J. Kamoun S. Raffaele S. (2012). Using hierarchical clustering of secreted protein families to classify and rank candidate effectors of rust fungi. PLoS One 7, e29847. 10.1371/journal.pone.0029847 22238666 PMC3253089

[B34] Sohn K. H. Lei R. Nemri A. Jones J. D. (2007). The downy mildew effector proteins ATR1 and ATR13 promote disease susceptibility in *Arabidopsis thaliana* . Plant Cell 19, 4077–4090. 10.1105/tpc.107.054262 18165328 PMC2217653

[B35] Souza T. L. P. O. Alzate-Marin A. L. Faleiro F. G. De Barros E. G. (2008). Pathosystem common bean—*Uromyces appendiculatus*: host resistance, pathogen specialization, and breeding for rust resistance. Pest Technol. 2, 56–69.

[B36] Sperschneider J. Dodds P. N. Gardiner D. M. Singh K. B. Taylor J. M. (2018). Improved prediction of fungal effector proteins from secretomes with EffectorP 2.0. Mol. Plant Pathol. 19, 2094–2110. 10.1111/mpp.12682 29569316 PMC6638006

[B37] Sperschneider J. Gardiner D. M. Dodds P. N. Tini F. Covarelli L. Singh K. B. (2016). EffectorP: predicting fungal effector proteins from secretomes using machine learning. New Phytol. 210, 743–761. 10.1111/nph.13794 26680733

[B38] Thomas W. J. Thireault C. A. Kimbrel J. A. Chang J. H. (2009). Recombineering and stable integration of the *Pseudomonas syringae* pv. *syringae* 61 hrp/hrc cluster into the genome of the soil bacterium *Pseudomonas fluorescens* Pf0-1. Plant J. 60, 919–928. 10.1111/j.1365-313X.2009.03998.x 19682294

[B39] Tyler B. M. Tripathy S. Zhang X. Dehal P. Jiang R. H. Aerts A. (2006). *Phytophthora* genome sequences uncover evolutionary origins and mechanisms of pathogenesis. Science 313, 1261–1266. 10.1126/science.1128796 16946064

[B40] Untergasser A. Cutcutache I. Koressaar T. Ye J. Faircloth B. C. Remm M. (2012). Primer3—new capabilities and interfaces. Nucleic Acids Res. 40, e115. 10.1093/nar/gks596 22730293 PMC3424584

[B41] Upadhyaya N. M. Mago R. Staskawicz B. J. Ayliffe M. A. Ellis J. G. Dodds P. N. (2014). A bacterial type III secretion assay for delivery of fungal effector proteins into wheat. Mol. Plant-Microbe Interact. 27, 255–264. 10.1094/MPMI-07-13-0187-FI 24156769

[B42] Ve T. Williams S. J. Catanzariti A. M. Rafiqi M. Rahman M. Ellis J. G. (2013). Structures of the flax-rust effector AvrM reveal insights into the molecular basis of plant-cell entry and effector-triggered immunity. Proc. Natl. Acad. Sci. U. S. A. 110, 17594–17599. 10.1073/pnas.1307614110 24101475 PMC3808616

[B43] Wan L. Koeck M. Williams S. J. Ashton A. R. Lawrence G. J. Sakakibara H. (2019). Structural and functional insights into the modulation of the activity of a flax cytokinin oxidase by flax rust effector AvrL567-A. Mol. Plant Pathol. 20, 211–222. 10.1111/mpp.12749 30242946 PMC6637871

[B44] Wang C. I. Guncar G. Forwood J. K. Teh T. Catanzariti A. M. Lawrence G. J. (2007). Crystal structures of flax rust avirulence proteins AvrL567-A and -D reveal details of the structural basis for flax disease resistance specificity. Plant Cell 19, 2898–2912. 10.1105/tpc.107.053611 17873095 PMC2048696

[B45] Wei C. F. Kvitko B. H. Shimizu R. Crabill E. Alfano J. R. Lin N. C. (2007). A *Pseudomonas syringae* pv. *tomato* DC3000 mutant lacking the type III effector HopQ1-1 is able to cause disease in the model plant *Nicotiana benthamiana* . Plant J. 51, 32–46. 10.1111/j.1365-313X.2007.03126.x 17559511

[B46] Yin C. Ramachandran S. R. Zhai Y. Bu C. Pappu H. R. Hulbert S. H. (2019). A novel fungal effector from *Puccinia graminis* suppressing RNA silencing and plant defense responses. New Phytol. 222, 1561–1572. 10.1111/nph.15676 30623449

[B47] Zha H. Fisk H. A. Yaffe M. P. Mahajan N. Herman B. Reed J. C. (1996). Structure-function comparisons of the proapoptotic protein Bax in yeast and mammalian cells. Mol. Cell. Biol. 16, 6494–6508. 10.1128/MCB.16.11.6494 8887678 PMC231651

