# Supplementary material for: Candidate Effectors From Uromyces appendiculatus, the Causal Agent of Rust on Common Bean, Can Be Discriminated Based on Suppression of Immune Responses
Source: Front Plant Sci. 2019 Oct 4;10:1182. doi: 10.3389/fpls.2019.01182 (PMC6787271; doi:10.3389/fpls.2019.01182)
Supplement: Supplementary file 2 [file Table_1.docx]

**Table S1. Primers for cloning Uaca_Ns.**

| Gene | Nested PCR outer primers | | Nested PCR inner primers | | Primers for re-cloning without signal peptide | |
| --- | --- | --- | --- | --- | --- | --- |
|  | Name | Sequence | Name | Sequence | Name | Sequence |
| Uaca_1 | Ua_RTP1f1  Ua_RTP1r1 | CATTCATTATGTTATTCAACCC  TCTGGTGATAGTTGTGGGTATC | Ua_RTP1f2zta  Ua_RTP1r2zta | ATGTTATTCAACCCGAATCTCC  TCATTCAGGAATGATGAAATCC | UaCA1nsf  UaCA1rT | ATGCACGTCACAGGCCCGG  TCATTCAGGAATGATGAAATCCCGATC |
| Uaca_2 | Ua_RTP2f1  Ua_RTP2r1 | AGAATAGCAAAATGTTTCTCCG  ATGACTGTGAGCCGTGGGAATC | Ua_RTP2f2zta  Ua_RTP2r2zta | ATGTTTCTCCGCCGAATTCAAG  TCACGACGGGGAGATTCTTTG | UaCA2nsf  UaCA2rT | ATGTCAAACCTCATCCCGATGAC  TCACGACGGGGAGATTCTTT |
| Uaca_3 | Ua_contig00519f1  Ua_contig00519r1 | AATAACGATCTCTAAATCATGC  GACGATTGGGTTCGACTAGCCG | Ua_contig00519f2zta  Ua_contig00519r2zta | ATGCGCTCTTTTGTCATCTGTG  CTAGCCGAGGTCACAGCC | UaCA3nsf  UaCA3rT | ATGCACCCCCTTGCCAAACAG  CTAGCCGAGGTCACAGC |
| Uaca_4 | Ua_contig08771f1  Ua_contig08771r1 | CACATTCAAACACTTAAAATGC  TGTCATAAGAGGTGATCATGGG | Ua_contig08771f2zta  Ua_contig08771r2zta | ATGCAATTCAACTATTTAGCCAG  TCATGGGCCTTGGAAAGTCTTAAG | UaCA4nsf  UaCA4rT | ATGCAAGCTCCACCTGCACCT  TCATGGGCCTTGGAAAGTCTTA |
| Uaca_5 | Ua_contig09006f2  Ua_contig09006r2 | TTAATCTTGGTTGAAACAATGG  TCTCTTGCTTTGAAGGCTGATC | Ua_contig09006f2zta  Ua_contig09006r2zta | ATGGTCCCTAGCATGATTTGG  TCAATTGCACCTCTTAGGCC | UaCA5nsf  UaCA5rT | ATGAACTGGGACCCCGCCA  TCAATTGCACCTCTTAGGCC |
| Uaca_7 | Ua_contig14788f1  Ua_contig14788r1 | CGACAAGCGATCCCATTCTC  ACGAATAGAAGTAAGAGGTGG | Ua_contig14788f2  Ua_contig14788r2 | ATGCATCTAGCTTCATTCTTG  TCAAGGGCCTTGCAAAGCTG | UaCA7nsf  UaCA7rT | ATGCAAAGCAACAACACGACACT  TCAAGGGCCTTGCAAAGC |
| Uaca_9 | Ua_contig01113f1  Ua_contig01113r1 | GTCAGACCCGAATTCCCAG  CGAGGACAACAATGGACGAG | Ua_contig01113f2  Ua_contig01113r2 | ATGAATACCGCACTTTTCGCTC  TCAAGAAGGCTTGAACCATGTG | UaCA9nsf  UaCA9rT | ATGGAAGGCGAAACTAGGCAATG  TCAAGAAGGCTTGAACCATG |
| Uaca_10 | Ua_contig08392f1  Ua_contig08392r1 | CAAGTTATCTCTCCCTTCAAAG  GAAGATTGAGTCCGGTGATG | Ua_contig08392f2  Ua_contig08392r2 | ATGGCCAACCTCGTTATTTTC  TCAACCAACAAGAGCACTTG | UaCA10nsf  UaCA10rT | ATGAAGTTCATACCTTCAGCGCA  TCAACCAACAAGAGCACTTG |
| Uaca_11 | Ua_contig09210f1  Ua_contig09210r1 | CACTCGTTCATCAATTAATCAC  GAAGTGTGGGTGCTCCGG | Ua_contig09210f2  Ua_contig09210r2 | ATGCAATCCTTTACACTCATGAC  TCATTTGAAGCCAGCCGTGC | UaCA11nsf  UaCA11rT | ATGGTGCAAGTTGAACCCCGA  TCATTTGAAGCCAGCCGT |
| Uaca_12 | Ua_contig16788f1  Ua_contig16788r1 | GTTGCCAACTTGTGACACTC  AGGAAGGTAGTGCATTGCTG | Ua_contig16788f2  Ua_contig16788r2 | ATGCAATCCTTTATACTCATGAC  TCATTTGAAACCAACCATGCTG | UaCA12nsf  UaCA12rT | ATGTACTCTGTACAAGTTGAACCCC  TCATTTGAAACCAACCATGC |
| Uaca_14 | Ua_contig08587f1  Ua_contig08587r1 | GCACCACAAACGTAAAAAAATG  GCTCAGGATAAAAACGGGTC | Ua_contig08587f2  Ua_contig08587r2 | ATGATGAAAGCTGTCTTACTTAG  TTAAACTGGAGCTTGTGCAGAG | UaCA14nsf  UaCA14rT | ATGGTGTCGGCAACACAGCAT  TTAAACTGGAGCTTGTGCAGA |
| Uaca_16 | Ua_contig13509f1  Ua_contig13509r1 | CATCTGGTGTATATCTATGACTC  GGTAACATGAGTAATAGAGCTAG | Ua_contig13509f2  Ua_contig13509r2 | ATGAAGACCTCGATGGTTTTG  TTAGTAGGGGTAGCGACGG | UaCA16nsf  UaCA16rT | ATGCCTGCCCAGGCAG  TTAGTAGGGGTAGCGACGGTA |
| Uaca_20 | Ua_contig07242f1  Ua_contig07242r1 | GATTGTCTGTCTCACTGTACC  CTAAGAAACCAGCGGCTATCG | Ua_contig07242f2  Ua_contig07242r2 | ATGTTTTCGAAAATAGCATTTGC  TCAAGCAGGTAGACCAGCTC | UaCA20nsf  UaCA20rT | ATGACCGCCATAGCATTCCCA  TCAAGCAGGTAGACCAGCTC |
| Uaca_22 | Uaca_22f1  Uaca_22r1 | CACTACCTCCACTCCACTCTC  GTAATATAGGAGGCAAGACAAAG | Uaca_22f2  Uaca_22r2 | ATGTTGAACCCATCCTGCAC  TTATGCTATAACAGCCACCAG | UaCA22nsf  UaCA22rT | ATGTCTAACACTAATGATACCACCGC  TTATGCTATAACAGCCACCAGC |
| Uaca_23 | Uaca_23f1  Uaca_23r1 | GATCGATACCACTTAGCCACTG  CTTGGCCTTGATCTAATGGGAG | Uaca_23f2  Uaca_23r2 | ATGCAGTTATTACAGGACTGG  TTACTGCTTCTTAGCAAACGC | UaCA23nsf  UaCA23rT | ATGTCTCAAGATGAATTCCCAGC  TTACTGCTTCTTAGCAAACGCA |
| Uaca_24 | Uaca_24f1  Uaca_24r1 | CATTGTACGACCGTTCCTTC  GGGAAGCAAGACCAGGAATG | Uaca_24f2  Uaca_24r2 | ATGCGTGCTACTACCATTGTTG  TTAAGCCTTTTCAGCATGCTTATC | UaCA24nsf  UaCA24rT | ATGGCTGACAACTCGGAGCAA  TTAAGCCTTTTCAGCATGCTT |
| Uaca_25 | Uaca_25f1  Uaca_25r1 | ATAATCACACACACAGGTATAC  GTGGTCTTTGGGAGTTTAATTC | Uaca_25f2  Uaca_25r2 | ATGGTTTCTTCAACGCAATTGG  TTAACAAGCGGTATGGGACG | UaCA25nsf  UaCA25rT | ATGCAAGAAAAGGCCGCCGATTC  TTAACAAGCGGTATGGGACG |
| Uaca_27 | Uaca_27f1  Uaca_27r1 | CCAAAGACAACAAGCTCCTG  CAGCATCTCCTCGGTCTCC | Uaca_27f2  Uaca_27r2 | ATGCGTTTCATAATCTGTGCAATC  TTAGGAATGCGGACCGCTC | UaCA27nsf  UaCA27rT | ATGCATCCCCTCTCCCAACAGTT  TTAGGAATGCGGACCGCTCA |
| Uaca_28 | Uaca_28f1  Uaca_28r1 | CCACCAGCGCACACAGTTG  GTGAGCGAATTTAGGCCATC | Uaca_28f2  Uaca_28r2 | ATGCGTGTCTCATGTGCTTTTATTTTATTTG  CTAAGATCGGGAGGCGAGTC | UaCA28nsf  UaCA28rT | ATGGCTCCAATTAGCTCCTCAAACG  CTAAGATCGGGAGGCGAGT |
| Uaca_31 | Uaca_31f1  Uaca_31r1 | CACACCACACACACAAACAC  CAAAGTGAGCGCATTCAATTAC | Uaca_31f2  Uaca_31r2 | ATGCGTTGCTCAGGAGTAATTATG  TTAAGCTATAAATGAGCCAAAAAAG | UaCA31nsf  UaCA31rT | ATGAAACCAATCGAGCATGACTTGG  TTAAGCTATAAATGAGCCAAAAAAGGCC |
| Uaca_32 | Uaca_32f1  Uaca_32r1 | CTCACAACTCCCTTATCTCAAC  GATGAGTTACTCAAGCTTACC | Uaca_32f2  Uaca_32r2 | ATGGCACACTTTCCCATTCG  TCATTCCTTTGAGTATTCAAATTTG | UaCA32nsf  UaCA32rT | ATGTCGATCAAGTGTTGTCCAGGA  TCATTCCTTTGAGTATTCAAATTTGGC |
| Uaca_34 | Uaca_34f1  Uaca_34r1 | CTAGTACAAGTTCACCAAACC  CATAGTGACCTGCATCAACAAC | Uaca_34f2  Uaca_34r2 | ATGCGCTTCTCTCTTACCTG  CTAGTAGTGGTCTTTATCGTC | UaCA34nsf  UaCA34rT | ATGGGTCCACTCTTGACTTCGGG  CTAGTAGTGGTCTTTATCGTCATCAT |
| Uaca_36 | Uaca_36f1  Uaca_36r1 | GGGTCGCTATTTGGCTCGG  GCCACCCATGCCAGCTTTC | Uaca_36f2  Uaca_36r2 | ATGCGCGCTCACCTGCAC  TCAACTGGCCCAGGGTTGC | UaCA36nsf  UaCA36rT | ATGTTGCCAGAGTTCCCTGAGTC  TCAACTGGCCCAGGGTT |
| Uaca_37 | Uaca_37f1  Uaca_37r1 | GCCTTCAATATTCTCTTCTGG  CTGTTACCCTTTTTATCTCTTTC | Uaca_37f2  Uaca_37r2 | ATGTTTTCTCGTCTTCTCAGC  TCAACTTAAATGGCCACCCC | UaCA37nsf  UaCA37rT | ATGAGGGTCGACCTTGGAGGT  TCAACTTAAATGGCCACCCC |
| Uaca_38 | Uaca_38f1  Uaca_38r1 | CTAAACGGACGAATCATCAAAG  GGTTTAACCCGAGAAAAATGC | Uaca_38f2  Uaca_38r2 | ATGCTCATTAGCTTTGCACTC  TTATAGACCGGGACAAGACTTTG | UaCA38nsf  UaCA38rT | ATGAGACGTCACCAATCTACACCC  TTATAGACCGGGACAAGACTTTGG |
| Uaca_40 | Uaca_40f1  Uaca_40r1 | CAGATTTACACATTAGCACTTC  CGGTTTCATGGTAGGACGAG | Uaca_40f2  Uaca_40r2 | ATGATGGTTCCGGCTGAAC  TTAGTTCCAGGCCAACCAAG | UaCA40nsf  UaCA40rT | ATGTCCGAGCCGACCAATGG  TTAGTTCCAGGCCAACCAAG |
| Uaca_41 | Uaca_41f1  Uaca_41r1 | TCTACCAATCCATTCAACAGTC  CCATGCTGAGTTGTCCTCC | Uaca_41f2  Uaca_41r2 | ATGTTATTCAAATATTTGATCGGAAC  CATTTGTAGAAGCTAATCGCTAC | UaCA41nsf  UaCA41rT | ATGCACCCAACGGCATCTTCG  CTAATCGCTACCAGAATAGAATGTGAT |
| Uaca_43 | Uaca_43f1  Uaca_43r1 | GCACCGCGTTTCCTTTCCTC  GTTCATGGGGTGTTAGTTTATC | Uaca_43f2  Uaca_43r2 | ATGAATATTCCACTTTTTTCGTC  TTAAAGAGATTTAGGTTTGGGAAAG | UaCA43nsf  UaCA43rT | ATGGATGTCCAATTCCAATGCCAAG  TTAAAGAGATTTAGGTTTGGGAAAGC |
| Uaca_44 | Uaca_44f1  Uaca_44r1 | CAGCATCAGTCAACGCCTCG  GAATCTCTTGTGTATATGTCATG | Uaca_44f2  Uaca_44r2 | ATGATCTCTCTTCGATCGCG  TTACCACGCTGCGCCTCGAG | UaCA44nsf  UaCA44rT | ATGGCTGCAATCCAAACGATTCC  TTACCACGCTGCGCCTC |
| Uaca_45 | Uaca_45f1  Uaca_45r1 | CTTCTACATTGACGCTATTACC  CCAGCACGGCCTTATCATC | Uaca_45f2  Uaca_45r2 | ATGCTTACCAGAAAACTTTCAAAC  TCATGATAATGCTGCTTTGC | UaCA45nsf  UaCA45rT | ATGCAAACCAACCCGAAGAATCCAA  TCATGATAATGCTGCTTTGCAAAA |
| Uaca_46 | Uaca_46f1  Uaca_46r1 | CTTGCCAACTTTGTCCGCG  GAGTTTGCTTCGCTGATATTATG | Uaca_46f2  Uaca_46r2 | ATGCGCACTCAACATGAAGC  CTATACCGATTTTTCTTTTTTGG | UaCA46nsf  UaCA46rT | ATGTCTAGCTTGTGGAAAAGAATGGAT  CTATACCGATTTTTCTTTTTTGGAATACTTT |
| Uaca_47 | Uaca_47f1  Uaca_47r1 | CACAATCTACTCGACAAGCTG  GTTGCTCGCCAGTGCTAGATC | Uaca_47f2  Uaca_47r2 | ATGAGCATGGATCGATTGGG  CTACCCGTAGTTGGTTAAAGG | UaCA47nsf  UaCA47rT | ATGGCAATTAACGTCGCAACCGA  CTACCCGTAGTTGGTTAAAGGAC |
