# Supplementary material for: Candidate Effectors From Uromyces appendiculatus, the Causal Agent of Rust on Common Bean, Can Be Discriminated Based on Suppression of Immune Responses
Source: Front Plant Sci. 2019 Oct 4;10:1182. doi: 10.3389/fpls.2019.01182 (PMC6787271; doi:10.3389/fpls.2019.01182)
Supplement: Supplementary file 3 [file Table_2.docx]

**Table S2. Primers for RT-qPCR.** Primers designed and efficiency tested as described in 2.9. The same method was also used in designing and testing the primers for the reference genes *Act* and *CytB*.

| **Gene** | **Primer names** | **Primer sequence 5’-3’** | **Amplicon length** | **Efficiency** |
| --- | --- | --- | --- | --- |
| *Uaca_2* | *Uaca_2_qPCR_1f*  *Uaca_2_qPCR_1r* | TTG GAG TGG TAC GTG TGC TG  AGA CTG ATG CCA ACC CCT TC | 200 | 94.5 |
| *Uaca_3* | *Uaca_3_qPCR_1f*  *Uaca_3_qPCR_1r* | CCT TGC CAA ACA GTC GAT CG  TCT GTA ATC CCA CGC CAA GG | 244 | 89.6 |
| *Uaca_4* | *Uaca_4_qPCR_2f*  *Uaca_4_qPCR_2r* | ACA CCC GCA CCC AAT ACA C  AGC CTC TGT TGG AAT CGT GG | 199 | 100.0 |
| *Uaca_5* | *Uaca_5_qPCR_2f*  *Uaca_5_qPCR_2r* | TTG GCT TGC TTT ATG GCT CC  ACA GTT GAA CCT CGG GAA AG | 183 | 98.9 |
| *Uaca_7* | *Uaca_7_qPCR_2f*  *Uaca_7_qPCR_2r* | CCC CCA AAA GAT CCG CAA AC  CTG TGG ACT CGA GCA TGA CC | 210 | 95.2 |
| *Uaca_9* | *Uaca_9_qPCR_2f*  *Uaca_9_qPCR_2r* | GCT CTG TTT TCA CTC GTC GC  AGG GTG CAG TTT GTG GCG G | 172 | 95.6 |
| *Uaca_10* | *Uaca_10_qPCR_1f*  *Uaca_10_qPCR_1r* | AGC TCT GTG GTT CCT ACG TG  ACC CGA CTT TGC ACC CTT AC | 237 | 99.8 |
| *Uaca_11* | *Uaca_11_qPCR_2f*  *Uaca_11_qPCR_2r* | GCT CAG TCA TCC TCA TCC TC  GAA GTG GTC TGC GAT ATG GC | 186 | 102.9 |
| *Uaca_12* | *Uaca_12_qPCR_2f*  *Uaca_12_qPCR_2r* | TCT CGG TGG TGG TAT GAA TG  GTG GTC TGC GAT ATG GCT TG | 233 | 81.8 |
| *Uaca_14* | *Uaca_14_qPCR_1f*  *Uaca_14_qPCR_1r* | TGG GTT AAT GGC GCA AAG AC  CAC GTT ATG CCT TGG TCG AG | 209 | 97.0 |
| *Uaca_16* | *Uaca_16_qPCR_1f*  *Uaca_16_qPCR_1r* | CAG GTG CCA CTA AAC AAG CG  GTA GGG GTA GCG ACG GTA AG | 189 | 96.7 |
| *Uaca_20* | *Uaca_20_qPCR_1f*  *Uaca_20_qPCR_1r* | TAT CAG TCC TGC AGT GCT TG  AAC TTT TGG AGA CGA GGG GC | 163 | 101.0 |
| *Uaca_22* | *Uaca_22_qPCR_1f*  *Uaca_22_qPCR_1r* | GGA TGT GCC GAG CTC TCT G  ACT GGG ACG AAG CAA CAC TC | 231 | 98.2 |
| *Uaca_23* | *Uaca_23_qPCR_1f*  *Uaca_23_qPCR_1r* | TTC TGC CTT TGT AGT CGA GC  CCG CCT GAT TAG TGG TCT TG | 202 | 93.6 |
| *Uaca_24* | *Uaca_24_qPCR_1f*  *Uaca_24_qPCR_1r* | GAC AAC TCG GAG CAA CAC AG  GGA GTC AGC GCT GTT AAC AC | 191 | 104.9 |
| *Uaca_28* | *Uaca_28_qPCR_1f*  *Uaca_28_qPCR_1r* | GAC TCG CTT GCC TCT ACA TC  ATG TAG AGG CAA GCG AGT CG | 157 | 94.2 |
| *Uaca_44* | *Uaca_44_qPCR_2f*  *Uaca_44_qPCR_2r* | CTG TCA TCT CAT CTC GCC GG  ATG GGA CCG CAG AGT TTA GC | 166 | 100.0 |
| *Act* | *UaActf*  *UaActr* | ACT TGA TCT TGC CGG TCG AG  CGG CAG TGG TGG TAA AGC TA | 82 | 98.2 |
| *CytB* | *UaCytBf*  *UaCytBr* | CCA GTT TCG ATC GTA CCG GA  CCG GTA TGG CTA GCA GGA TT | 124 | 98.0 |
