# Supplementary figures and images for: Candidate Effectors From Uromyces appendiculatus, the Causal Agent of Rust on Common Bean, Can Be Discriminated Based on Suppression of Immune Responses
Source: Front Plant Sci. 2019 Oct 4;10:1182. doi: 10.3389/fpls.2019.01182 (PMC6787271; doi:10.3389/fpls.2019.01182)

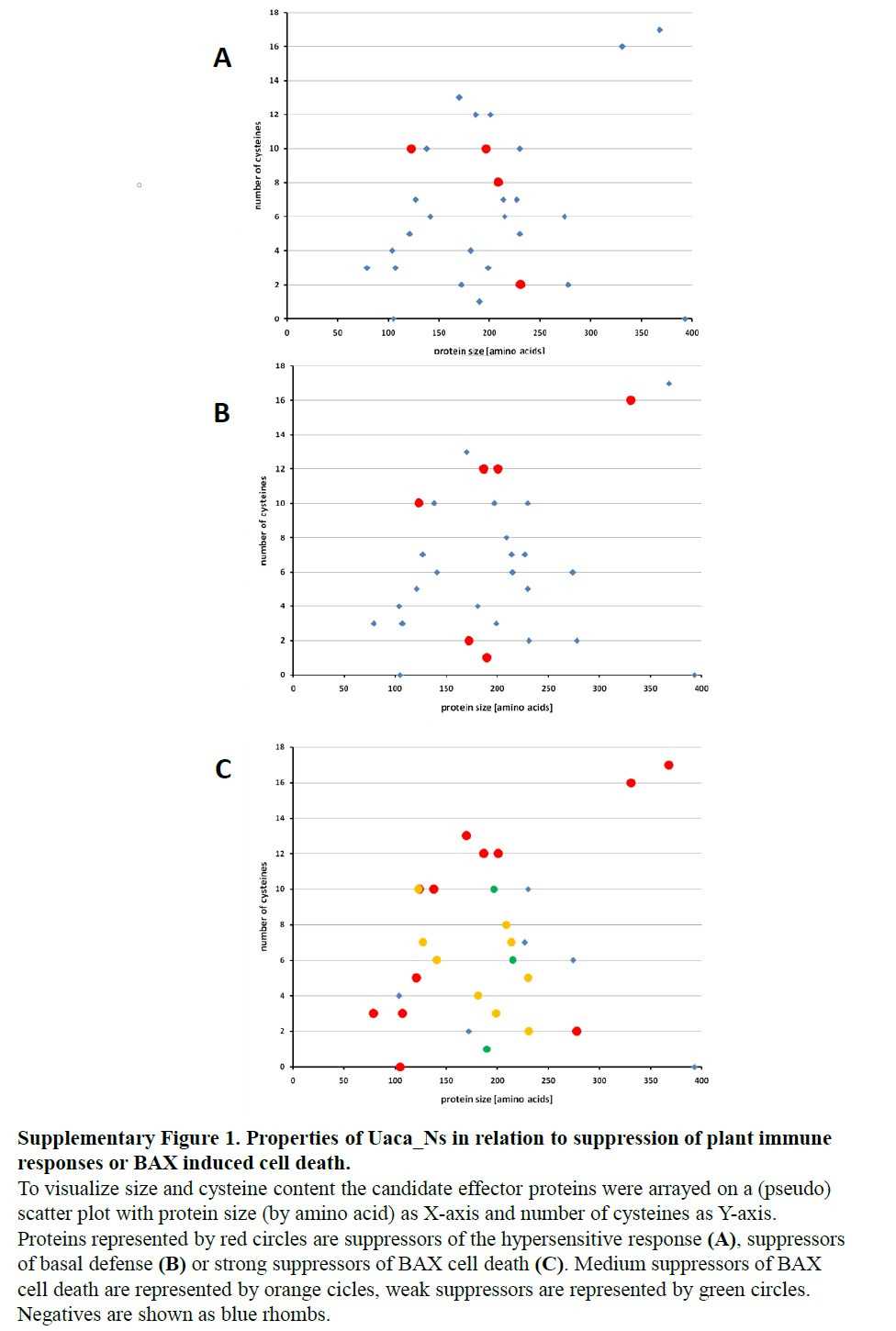

Supplement: Supplementary file 4 [file Image_1.jpg]

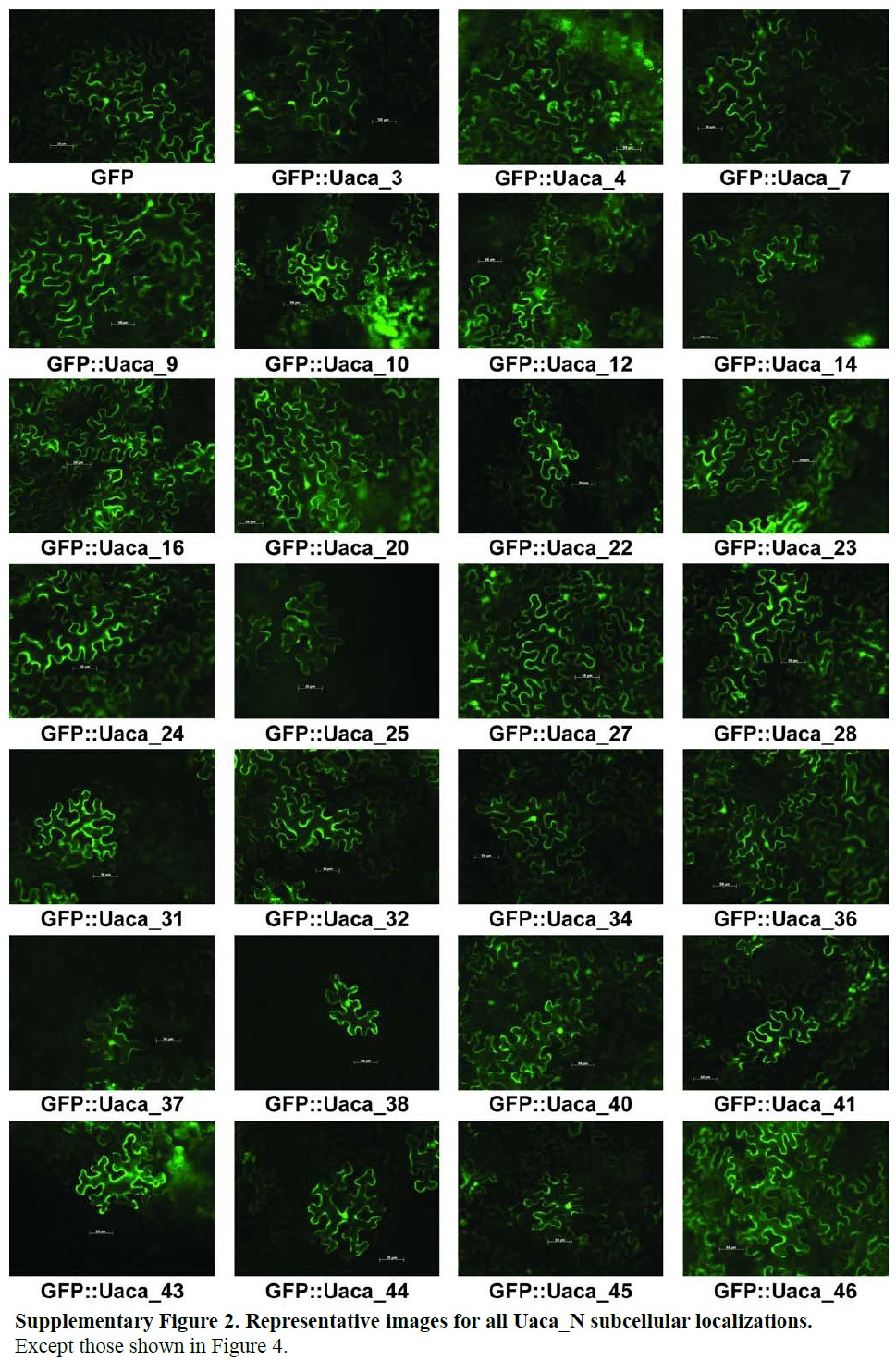

Supplement: Supplementary file 5 [file Image_2.jpg]

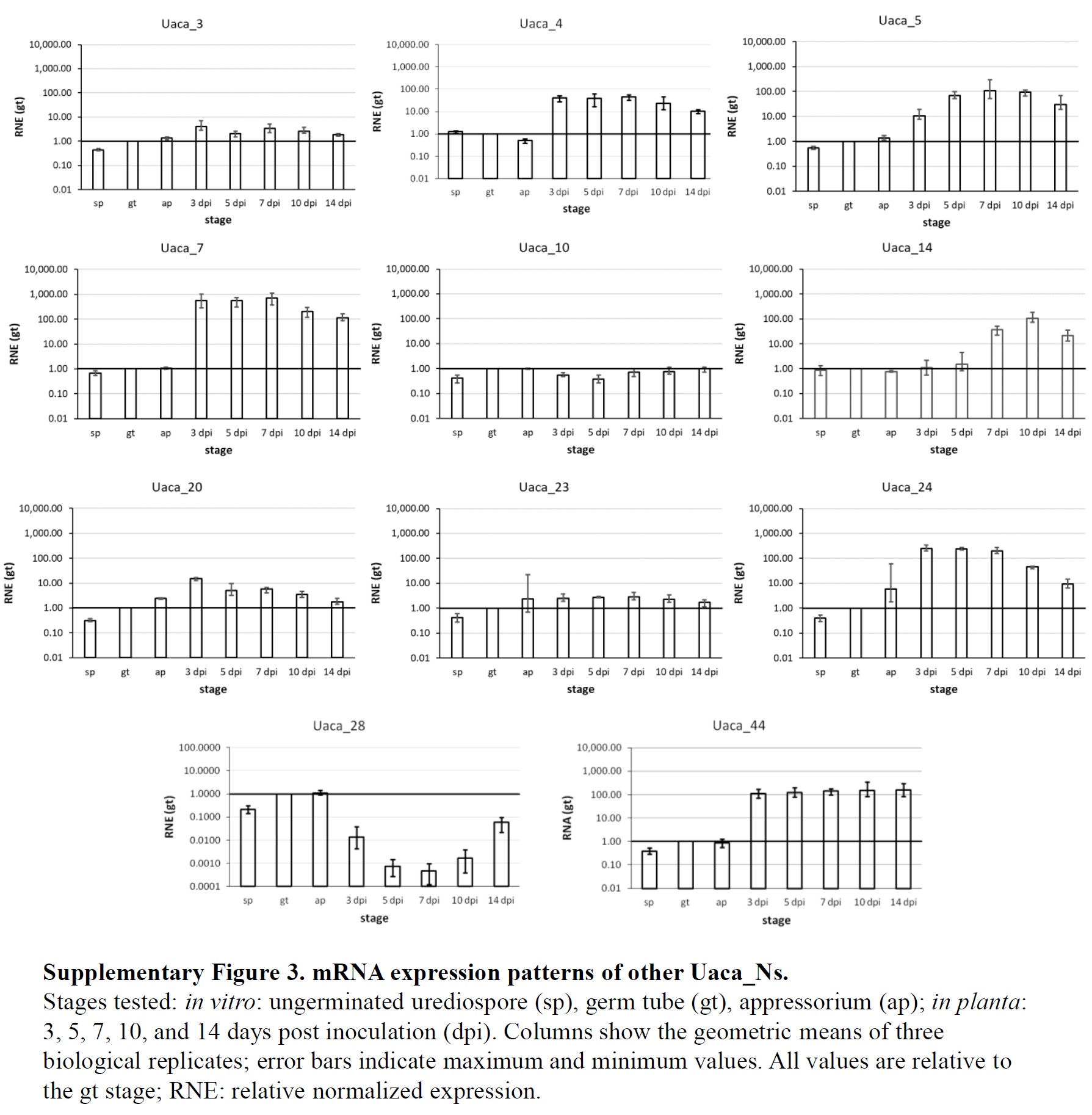

Supplement: Supplementary file 6 [file Image_3.jpg]
